# Probabilistic projection of the sex ratio at birth and missing female births by State and Union Territory in India

**DOI:** 10.1371/journal.pone.0236673

**Published:** 2020-08-19

**Authors:** Fengqing Chao, Christophe Z. Guilmoto, Samir K. C., Hernando Ombao

**Affiliations:** 1 Statistics Program, Computer, Electrical and Mathematical Sciences and Engineering Division, King Abdullah University of Science and Technology, Thuwal, Saudi Arabia; 2 CEPED/IRD, Université de Paris, Paris, France; 3 Asian Demographic Research Institute, Shanghai University, Shanghai, China; 4 Wittgenstein Centre for Demography and Global Human Capital (University of Vienna, IIASA, VID/OeAW), International Institute for Applied Systems Analysis, Laxenburg, Austria; Institute of Economic Growth, INDIA

## Abstract

The sex ratio at birth (SRB) in India has been reported to be imbalanced since the 1970s. Previous studies have shown there is a great variation in the SRB between geographic locations across India till 2016. Considering the enormous population and regional heterogeneity of India, producing probabilistic SRB projections at the state level is crucial for policy planning and population projection. In this paper, we implement a Bayesian hierarchical time series model to project the SRB across India by state. We generate SRB probabilistic projections from 2017 to 2030 for 29 States and Union Territories (UTs) in India, and present results for 21 States/UTs with data available from the Sample Registration System. Our analysis takes into account two state-specific factors that contribute to sex-selective abortion in India, resulting in sex imbalances at birth: the intensity of son preference and fertility squeeze. We project that the highest deficits in female births will occur in Uttar Pradesh, with a cumulative number of missing female births of 2.0 (95% credible interval [1.9; 2.2]) million from 2017 to 2030. The total female birth deficits during 2017–2030 for the whole of India is projected to be 6.8 [6.6; 7.0] million.

## Introduction

There has been a reported imbalance in India in the sex ratio at birth (SRB; defined as the ratio of male to female births) since the 1970s [1–7]. The masculinized SRB for India is a direct result of the practice of sex-selective abortions at the national level. However, unlike other countries similarly affected by sex imbalances at birth, India is unique in its regional diversity of SRB trajectories. Some states, such as Punjab, have experienced an early and rapid rise in birth masculinity since the 1980s, whereas in North Indian states, the masculinized SRB started to increase later. However, during the same period, many regions, most notably in South and East India, remained almost untouched by the emergence of prenatal sex selection seen in the rest of the country. Previous studies have shown significant variations in SRB levels and trends across geographic locations in India through the mid-2010s [7–11].

According to the UN, India is projected to become the world’s most populated country around the mid-2020s [12]. India consequently has a disproportionate influence on SRB global statistics. The diversity of India’s regional trajectories, therefore, requires a detailed, disaggregated approach for understanding the future development of sex imbalances at birth. Producing SRB projections for India at the state level is particularly crucial for appropriate population projection and policy planning. The absence of clear-cut trends in sex imbalances at birth warrants the use of a probabilistic methodology to project the future sex ratio at birth in India at the state level.

There are challenges in constructing state-level probabilistic SRB projections per Indian State and Union Territory because of specific unique characteristics of the country. As of 2017, India is divided into 36 States and Union Territories (UTs). First of all, an imbalanced SRB emerged in India in the mid-1970s, years before similar SRB imbalances became apparent in other countries [13]. Moreover, the SRB imbalance in India emerged even though its national total fertility rate was still close to five children per woman. In other countries, the rise in the SRB occurred at significantly lower fertility levels, closer to or below three children per woman. This observation implies that, all other things being equal, the preference for males in prenatal sex selection in India is stronger than in other countries affected by sex imbalances at birth. Hence, the mechanisms and rationale of the sex ratio transition experienced in other countries may not be entirely similar, and thus not directly applicable, to India’s situation. Secondly, state-level birth data both before and during the 1980s are scarce in India due to the lack of reliable birth registration systems. Even in the most recent decades, sources on sex imbalances at births are mostly limited to the Sample Registration System (SRS; an annual demographic panel household survey) and to the different rounds of the Demographic Health Surveys (DHS; called the National Family Health Survey in India). Given that the sample size from each Indian State/UT is much smaller than that for the entire country, there are more uncertainties associated with birth data at the state level than on the national level. The lack of detailed and informative birth data makes it difficult to study the pattern of the sex ratio transition experienced by Indian State/UT and prevents projections of state-level SRB trends solely based on state-level birth data.

Given the importance of projecting SRB at regional level to monitor sex imbalances at birth in India, there have been various discussions on this issue in previous studies [2, 6, 14, 15]. However, these projections have been based exclusively on expert opinion and assumptions or apply only to the country as a whole. Specifically, National Commission on Population (NCP) of India relied on linear extrapolation to project the state-level SRB [15]. The projected SRB published by the NCP reaches 1.100 for nine States/UTs and 1.060 for 13 States/UTs in 2031, conditioning on whether the state-specific SRB level during the period 2015–2017 is above or below 1.100. Furthermore, the projections by NCP did not take into account the potential effect from fertility transition on SRB imbalance.

To the best of our knowledge, this is the first study to offer probabilistic projections of the SRB for Indian States/UTs based on a reproducible modeling approach. We develop a Bayesian hierarchical model to construct state-specific SRB projections for the period 2017–2030. With the hierarchical model, we are able to draw information from the estimation period 1990–2016 and share it between data-rich state-years and those with limited or no data. Furthermore, the hierarchical model structure allows for capturing state-level differences in levels and trends when data so indicate. Our projection model also accounts for two out of the three main factors that contribute to sex-selective abortion and imbalanced SRB [4, 5]: (i) the intensity of son preference, approximated here by the desired sex ratio at birth (DSRB); and (ii) the “fertility squeeze” effect [5], approximated by the total fertility rate (TFR). The third major precondition for skewed SRBs, i.e. accessibility to technology, is not considered here because no annual estimate or projection is available by Indian State/UT for the period 1990–2030.

## Materials and methods

In this paper, we model and project the SRB in the largest 29 Indian States/UTs for which we have adequate data at our disposal. Note that Telangana is combined with Andhra Pradesh because it separated from Andhra Pradesh only in 2014. Hence, we use the name “former state of Andhra Pradesh” to refer to the combination of Andhra Pradesh and Telangana in our study. S1 Appendix includes all details on data preprocessing, model specifications, computing, post-model calculation, and we summarize them in this section. We present state-level results for 21 States/UTs that are included in the India Sample Registration System (SRS), covering 98.4% of the total population of India as of the year 2011. S1 Table lists the 29 Indian States/UTs included in our study and the 21 States/UTs for which we present the state-level results.

### Data

The SRB estimates by Indian State/UT from 1990 to 2016 are taken from [8]. The state-level covariates are either directly taken from external sources or are specifically modeled in this study. The covariate for approximating son preference intensity, the desired sex ratio at birth (DSRB), is estimated and projected for the period 1990–2035 using the Demographic and Health Surveys (DHS) data based on a Bayesian hierarchical model (explained in details below). The resulting DSRB estimates and projections used for the SRB model are presented in the S1 Appendix. The total fertility rate (TFR) data by Indian State/UT for 1990–2030 are available from the India Sample Registration System (SRS) and [16]. The projections of the number of births during 2017–2030 by Indian State/UT are from [16], which we used for computing the projected number of missing female births.

### Bayesian hierarchical model for state-level sex ratio at birth

The state-level SRB in India is modelled as the product of two components: 1) the baseline level of SRB; and 2) the state-year-specific multiplier. The baseline (or reference) SRB is assumed to be constant over time and the same for all States/UTs at the national level, as given by the India SRB baseline taken from [13]. The state-year-specific multiplier is modeled on the log-scale with an auto-regressive time series model of order 1, conditioned on a state-year-specific mean. For each state-year, the conditional mean of the time series model is expressed as a multivariate regression model with two covariates: (i) DSRB on the log scale; and (ii) TFR on the log scale.

Let *R*_*c*,*t*_ be the true SRB for an Indian State/UT *c* in year *t*. We model *R*_*c*,*t*_ on the log-scale and let *S*_*c*,*t*_ = log(*R*_*c*,*t*_). For the *i*-th SRB estimate on the log-scale *s*_*i*_ for Indian State/UT *c*[*i*] in year *t*[*i*], the model follows a normal distribution on the log-scale:

$$\begin{array}{c} s_{i}∼N\left(S_{c[i],t[i]},0.001^{2}\right),\;\text{for}\;i\in \left\{1,\cdots ,566\right\}. \end{array}$$ (1)

The mean of the distribution *S*_*c*,*t*_ for Indian States/UTs *c* ∈ {1, ⋯, *C*} and year *t* ∈ {1, ⋯, *T*} (where *t* = 1 refers to year 1990 and *t* = *T* refers to year 2030) is modeled as:

$$\begin{array}{c} S_{c,t}=\text{log}\left(N\right)+P_{c,t}, \end{array}$$ (2)

where *N* = 1.053 is the baseline level of SRB for the whole of India taken from [13].

The state-year-specific multiplier *P*_*c*,*t*_ accounts for the discrepancy of *S*_*c*,*t*_ from the log of national SRB baseline log(*N*). It is assumed to follow a time series model with AR(1) structure, conditioning on state-year-specific mean *V*_*c*,*t*_. For *c* ∈ {1, ⋯, *C*}:

$$\begin{array}{ccc} P_{c,t}|V_{c,t} & ∼ & N(V_{c,t},\sigma_{\epsilon }^{2}/\left(1-\rho_{c}^{2}\right)),\;\text{for}\;t=1, \end{array}$$ (3)

$$\begin{array}{ccc} P_{c,t}|P_{c,t-1},V_{c,t} & = & V_{c,t}+\rho_{c}\left(P_{c,t-1}-V_{c,t}\right)+\epsilon_{c,t},\;\text{for}\;t\in \left\{2,\cdots ,T\right\}, \end{array}$$ (4)

$$\begin{array}{ccc} \epsilon_{c,t} & ∼ & N\left(0,\tau_{\epsilon_{c}}^{-1}\right),\;\text{for}\;t\in \left\{2,\cdots ,T\right\}. \end{array}$$ (5)

*V*_*c*,*t*_ is modeled as a multivariate regression with two covariates: (i) *D*_*c*,*t*+5_: log of desired sex ratio at birth (DSRB), where the 5-year time lag in the regression model reflects the assumption that the DSRB generated from DHS of women under age 35 should represent the desire at the time before the first births [17]; (ii) *f*_*c*_(*F*_*c*,*t*_): state-specific non-linear function for the log of total fertility rate (TFR) *F*_*c*,*t*_.

$$\begin{array}{ccc} V_{c,t} & = & \alpha_{c}D_{c,t+5}+f_{c}(F_{c,t}),\;\text{for}\;t\in \left\{1,\cdots ,T\right\}. \end{array}$$ (6)

The state-specific coefficient parameters *α*_*c*_ for the covariate DSRB are modeled with hierarchical normal distributions in order to not only capture the differences across states, but also to exchange information between data-rich and data-poor States/UTs:

$$\begin{array}{ccc} \alpha_{c}|\tau_{\alpha } & \overset{\text{i}.\text{i}.\text{d}.}{∼} & N\left(0,\tau_{\alpha }^{-1}\right),\;\text{for}\;c\in \left\{1,\cdots ,C\right\}. \end{array}$$ (7)

The state-specific function *f*_*c*_(⋅) is a second-order random walk (RW2) model as a continuous time process [18] on the log-scaled TFR *F*_*c*,*t*_. The function is flexible to incorporate the non-linear fertility transition given the reverse of fertility at very low level [19]. The state-specific function *f*_*c*_(*F*_*c*,*t*_) is specified as:

$$\begin{array}{ccc} f_{c}(F_{c,t})=\Delta_{c,t}^{2} & = & F_{c,t}-2F_{c,t-1}+F_{c,t-2}, \end{array}$$ (8)

$$\begin{array}{ccc} \Delta_{c,t}^{2} & ∼ & N(0,\tau_{c}^{-1}). \end{array}$$ (9)

The state-specific auto-regressive parameter *ρ*_*c*_ and $\tau_{\epsilon_{c}}$ and the precision parameters *τ*_*α*_ and *τ*_*c*_ for the state-specific DSRB coefficient and RW2-transformed TFR are assigned with Penalized Complexity (PC) priors as explained in [20]. The densities of the PC priors are specified in S1 Appendix.

#### Bayesian hierarchical model for state-level desired sex ratio at birth

We develop a Bayesian hierarchical model to estimate and project DSRB, which is used as a model input for the SRB projection model as described previously. The state-specific DSRB exp{*D*_*c*,*t*_} for an Indian State/UT *c*, for year *t* is modeled as the sum of the reference level of DSRB and the distortion of DSRB away from the reference:

$$\begin{array}{c} \text{exp}\{D_{c,t}\}=1+\Delta_{c,t}. \end{array}$$ (10)

exp{*D*_*c*,*t*_} is modeled as a sum of two elements: (i) 1, indicating no preference between daughters and sons. We choose 1 instead of the baseline SRB value as the value which the DSRB will eventually converge to, because DSRB reflects the desire of women’s preference for their offspring composition, not the actual realization of the sex composition for live births; and (ii) Δ_*c*,*t*_, representing the level of son preference for State/UT *c* in year *t*.

The state-year-specific distortion of DSRB Δ_*c*,*t*_ is modeled as a scaled logistic function with independent variable log(*t*) log of time index, state-specific coefficient *ϕ*_*c*_ (for rate of decline) and intercept parameters *ζ*_*c*_ (for the average level), and the scale parameter *δ*_*c*_ which models the maximum DSRB on a state level. We use the scaled logistic function to model the general decline of son preference over time and to reflect that the rate of decline is slower when the son preference intensity is weaker. Hence, the model for Δ_*c*,*t*_ is:

$$\begin{array}{c} \Delta_{c,t}=\frac{\delta_{c}\cdot \text{exp}\{\phi_{c}\cdot \text{log}\left(t\right)+\zeta_{c}\}}{1+\text{exp}\{\phi_{c}\cdot \text{log}\left(t\right)+\zeta_{c}\}}. \end{array}$$ (11)

Normal hierarchical distributions are used for *δ*_*c*_, *ϕ*_*c*_ and *ζ*_*c*_ for *c* ∈ {1, ⋯, *C*}:

$$\begin{array}{ccc} \delta_{c} & ∼ & N(\mu_{\delta },\sigma_{\delta }^{2}), \end{array}$$ (12)

$$\begin{array}{ccc} \phi_{c} & ∼ & N(\mu_{\phi },\sigma_{\phi }^{2}), \end{array}$$ (13)

$$\begin{array}{ccc} \zeta_{c} & ∼ & N(\mu_{\zeta },\sigma_{\zeta }^{2}). \end{array}$$ (14)

The data quality model for DSRB takes into account the sampling error (reflecting a multi-stage stratified sampling design, with probability proportional to population size in the first stage and random selection of census enumeration block and/or households in the following stages) [21] and non-sampling error (indicating non-measurable errors like non-response, data input error, etc.). Vague priors are assigned to the hierarchical mean, the standard error parameters of the hierarchical distributions, and the non-sampling error parameters. The data quality model and priors are specified in the S1 Appendix.

### Estimates of missing female births and identifying Indian States/UTs with imbalanced SRB

We compute the annual number of missing female births (AMFB) to quantify the realization of SRB imbalance in a population due to sex-selective abortion for a certainty year. The AMFB is defined as the difference between the estimated and expected female live births for an Indian State/UT in a certain year. The estimated and expected female live births are computed based on the total births for a certain state-year (available from [16]) and the corresponding estimated and expected SRB, following the method as described in [22]. The cumulative effect of AMFB over time is referred as the cumulative number of missing female births (CMFB), which is defined as the sum of AMFB for a certain period.

An Indian State/UT is identified to have SRB imbalance if its AMFB in at least one year since 2017 is above zero for more than 95% of the posteriors samples.

### Model validation

For the 29 Indian States/UTs, we leave out data points, both state-level SRB median estimates from prior study [8] and covariates DSRB and TFR, after the year 2012. The left-out year is based on the availability of the state-level TFR data, i.e. 20% of the TFR are left out after 2012. After leaving out data, we fit the model to the training dataset, and obtain median estimates and credible intervals that would have been constructed based on available dataset in the left out year selected.

We calculate median errors and median absolute errors for the left-out observations. In this study, the left-out data are the state-level SRB median estimates after the year 2012. Error for the *j*-th left-out data is defined as:

$$\begin{array}{c} e_{j}=r_{j}-{\tilde{r}}_{j}, \end{array}$$ (15)

where ${\tilde{r}}_{j}$ refers to the posterior median of the predictive distribution based on the training data set for the *j*-th left-out SRB data *r*_*j*_. The coverage of 95% prediction interval is given by:

$$\begin{array}{c} {\text{Coverage}}_{95\%}=\frac{1}{J}\sum_{j=1}^{J}I_{j}\left(r_{j}\geq {{\text{ppd}}_{2.5\%}}_{j}\right)I_{j}\left(r_{j}\leq {{\text{ppd}}_{97.5\%}}_{j}\right), \end{array}$$ (16)

where *J* refers to the number of left-out SRB data. I(·)=1 if the condition in the brackets is true and I(·)=0 if the condition in the brackets is false. ppd_2.5%*j*_ and ppd_97.5%*j*_ correspond to the 2.5-th and 97.5-th percentiles of the posterior predictive distribution for the *j*-th left-out data *r*_*j*_. The coverage of 80% prediction interval is calculated in the same way by replacing ppd_2.5%*j*_ and ppd_97.5%*j*_ with ppd_10%*j*_ and ppd_90%*j*_ respectively. The validation measures are calculated for 1,000 sets of left-out data, where each set consists one randomly selected left-out SRB data from each State/UT. The reported validation results are based on the mean of the outcomes from the 1,000 sets of left-out data.

For the point estimates based on full data set and training data set, errors for the true level of SRB are defined as:

$$\begin{array}{c} e{\left(R\right)}_{c,t}={\hat{R}}_{c,t}-{\tilde{R}}_{c,t}, \end{array}$$ (17)

where ${\hat{R}}_{c,t}$ is the posterior median for State/UT *c* in year *t* based on the full data set, and ${\tilde{R}}_{c,t}$ is the posterior median for the same state-year based on the training data set. Coverage is computed in a similar manner as for the left-out observations, based on the lower and upper bounds of the 95% and 80% credible intervals of ${\tilde{R}}_{c,t}$ from the training data set.

## Results

### Covariate effect on state-level sex ratio at birth

**Fig 1:**
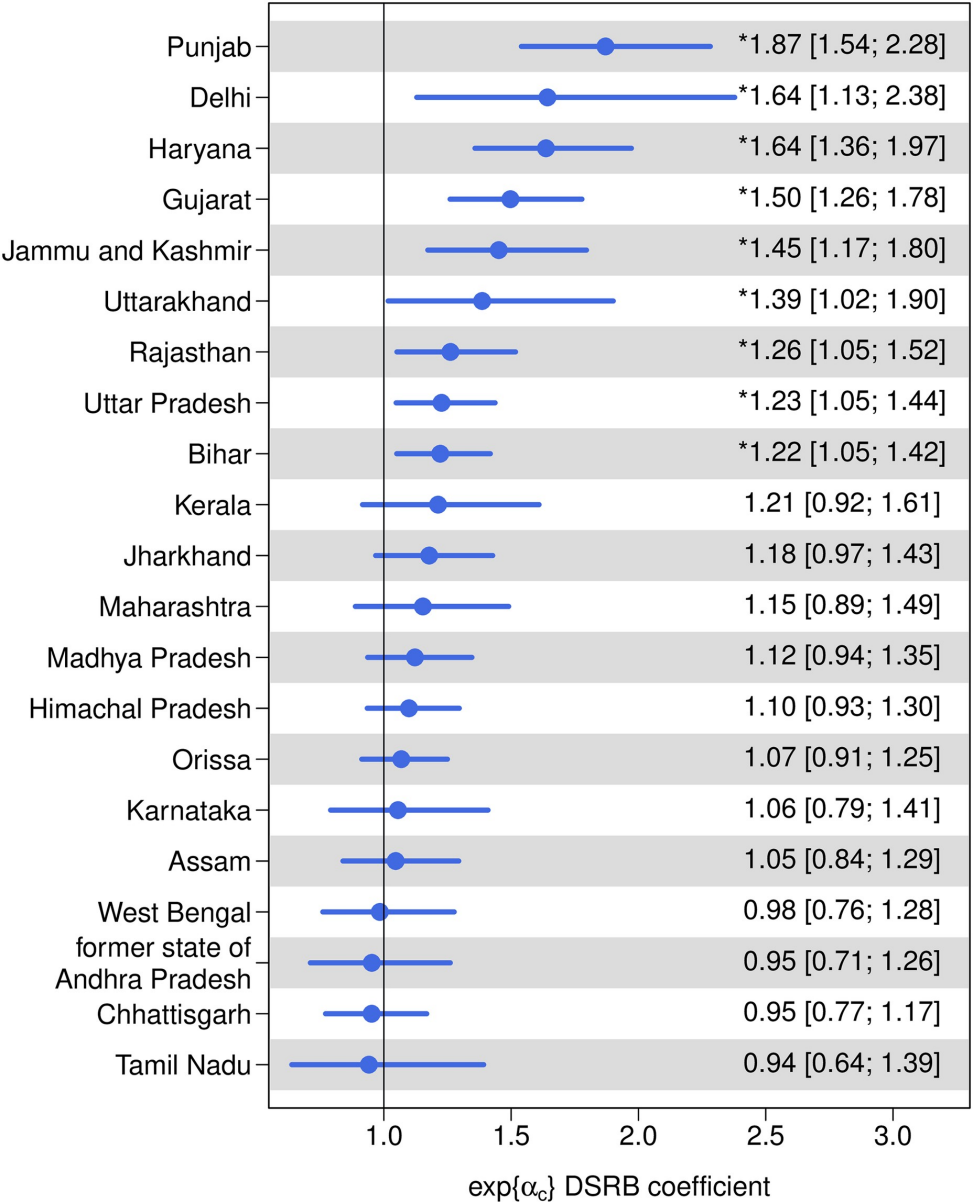
DSRB effect on SRB by Indian State/UT. Dots are median estimates. Horizontal line segments are 95% credible intervals. * indicates that the effect is statistically significantly different from 1. States/UTs are in descending order of the median estimates. Results are shown for the 21 Indian States/UTs with SRS data.

Fig 1 summarizes the effect of son preference intensity, using the desired sex ratio at birth (DSRB) as a proxy. Among the 21 Indian States/UTs with SRS data, 17 of them record a positive effect of son preference on the SRB, i.e., the exponential of the DSRB coefficient median estimates is bigger than 1 for 17 States/UTs. In other words, given all other covariates, when the son preference intensity (DSRB level) decreases over time, the SRB in these States/UTs will decrease. In particular, the effect of son preference is statistically significant in nine States/UTs (in the order of median estimates): Punjab with son preference effect at 1.87 (95% credible interval [1.54; 2.28]), Delhi at 1.64 [1.13; 2.38], Haryana at 1.64 [1.36; 1.97], Gujarat at 1.50 [1.26; 1.78], Jammu and Kashmir at 1.45 [1.17; 1.80], Uttarakhand at 1.39 [1.02; 1.90], Rajasthan at 1.26 [1.05; 1.52], Uttar Pradesh at 1.23 [1.05; 1.44], and Bihar at 1.22 [1.05; 1.42]. None of the States/UTs have a statistically significant negative son preference effect on SRB (i.e., less than 1).

**Fig 2:**
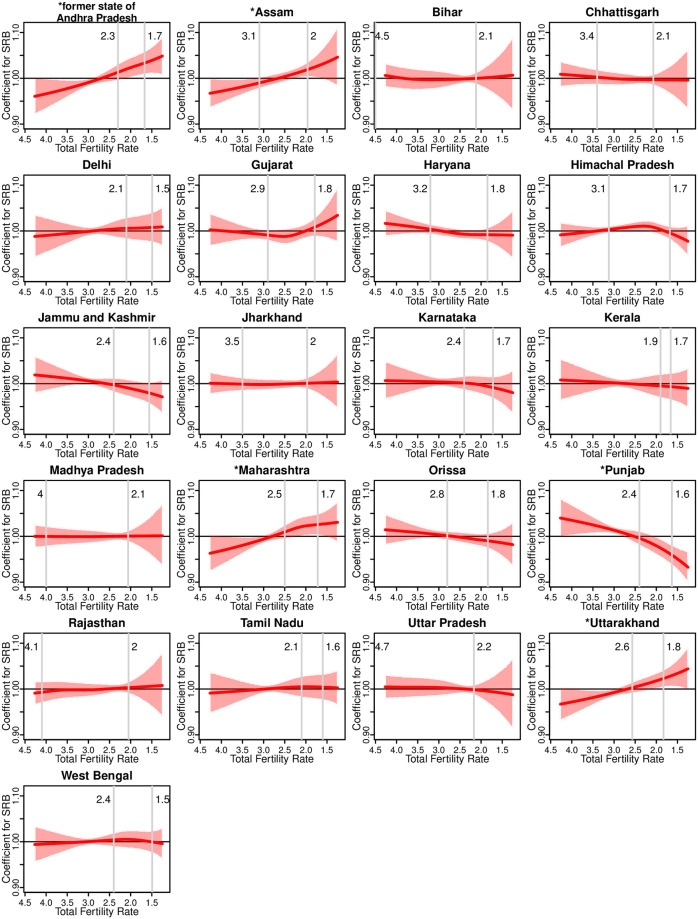
TFR effect on SRB by Indian State/UT. Curves are median estimates. Shaded areas are 95% credible intervals. The maximum and minimum values of TFR that is available for each State/UT during 1990–2030 are indicated with vertical lines and values are shown by the lines. * in front of a State/UT name indicates that the effect of TFR is statistically significantly different from 1 for at least one given value of TFR. Results are shown for the 21 Indian States/UTs with SRS data.

The effects of fertility decline on state-level SRB, represented by the TFR, are illustrated in Fig 2. The model results show that the effects of TFR on SRB differ in levels and trends across the different Indian States and UTs. These trends can be categorized into four groups: (i) monotonic increase as TFR decreases; (ii) monotonic decrease as TFR decreases; (iii) non-linear; (iv) no apparent effect, i.e., horizontal around 1. For the first type of trend, monotonic increases as TFR declines over time (except at very low TFR levels where a slight reverse usually occurs), the model suggests that the effect of TFR on SRB changes from negative (below 1) to positive (above 1) in four States/UTs. This category includes the former state of Andhra Pradesh (including Telangana), Assam, Maharashtra, and Uttarakhand, where the TFR effect on SRB is statistically different from 1 for at least one given TFR value. In general, for these four states, with a high TFR of above three children per woman, as the fertility declines, the SRB also declines, given other covariates fixed. When the TFR declines further to three and below, the SRB increases when the other covariates are fixed. In the second trend type with monotonic decreases, the effect of TFR on SRB changes from positive to negative in four States/UTs: Haryana, Jammu and Kashmir, Orissa, and Punjab. Compared to the first trend type, the decreasing trend of the TFR effect is much more gradual. Among the four States/UTs, the TFR effect is statistically different from 1 for at least one TFR value in Punjab only. The model suggests a non-linear relation between the effect on SRB and TFR in Gujarat and Himachal Pradesh.

### Sex ratio at birth projection for Indian States/UTs

**Fig 3:**
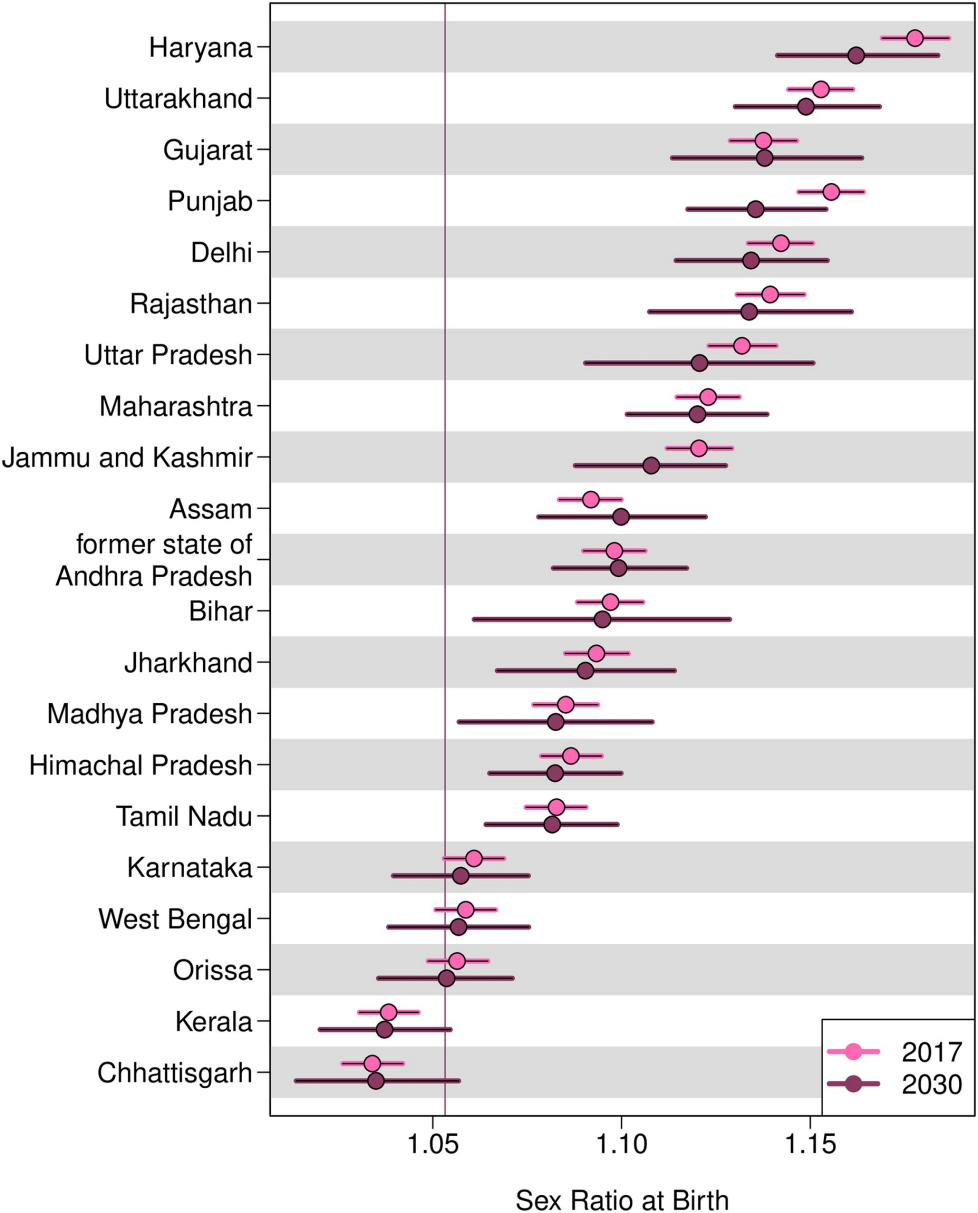
SRB projections by Indian state, in 2017 and 2030. Dots are median estimates. Horizontal line segments are 95% credible intervals. The horizontal line refers to the SRB baseline 1.053 for the whole India [13]. States/UTs are in descending order of the median projections of SRB in 2030. Results are presented for the 21 Indian States/UTs with SRS data.

The levels and trends in the SRB projections from 2017 to 2030 vary across Indian States/UTs (Fig 3). In 2030, the SRB ranges from 1.035 (95% credible interval [1.014; 1.057]) in Chhattisgarh and 1.037 [1.020; 1.055] in Kerala to 1.149 [1.130; 1.68] in Uttarakhand and 1.162 [1.141; 1.184] in Haryana. The SRB in 2030 is significantly higher than the national SRB baseline of 1.053 in 16 Indian States/UTs among the 21 Indian States/UTs for which we present results. In particular, the SRB in 2030 for six States/UTs is significantly above 1.100: in Haryana, in Uttarakhand, in Gujarat at 1.138 [1.113; 1.164], in Punjab at 1.136 [1.118; 1.154], in Delhi at 1.134 [1.114; 1.154], and in Rajasthan at 1.134 [1.107; 1.161].

During the period 2017–2030, the SRB median estimates in four Indian states are projected to increase: Assam (with the largest increase at 0.008 [-0.015; 0.032] from 2017 to 2030), the former state of Andhra Pradesh (including Telangana), Chhattisgarh, and Gujarat. None of the increases in these states are significantly different from zero. Among the 17 States/UTs show decreases in SRB median estimates during 2017–2030, four have median declines greater than -0.01: in Punjab at -0.020 [-0.040; 0.000], in Haryana at -0.015 [-0.039; 0.008], in Jammu and Kashmir at -0.013 [-0.034; 0.009], and in Uttar Pradesh at -0.011 [-0.042; 0.020].

**Fig 4:**
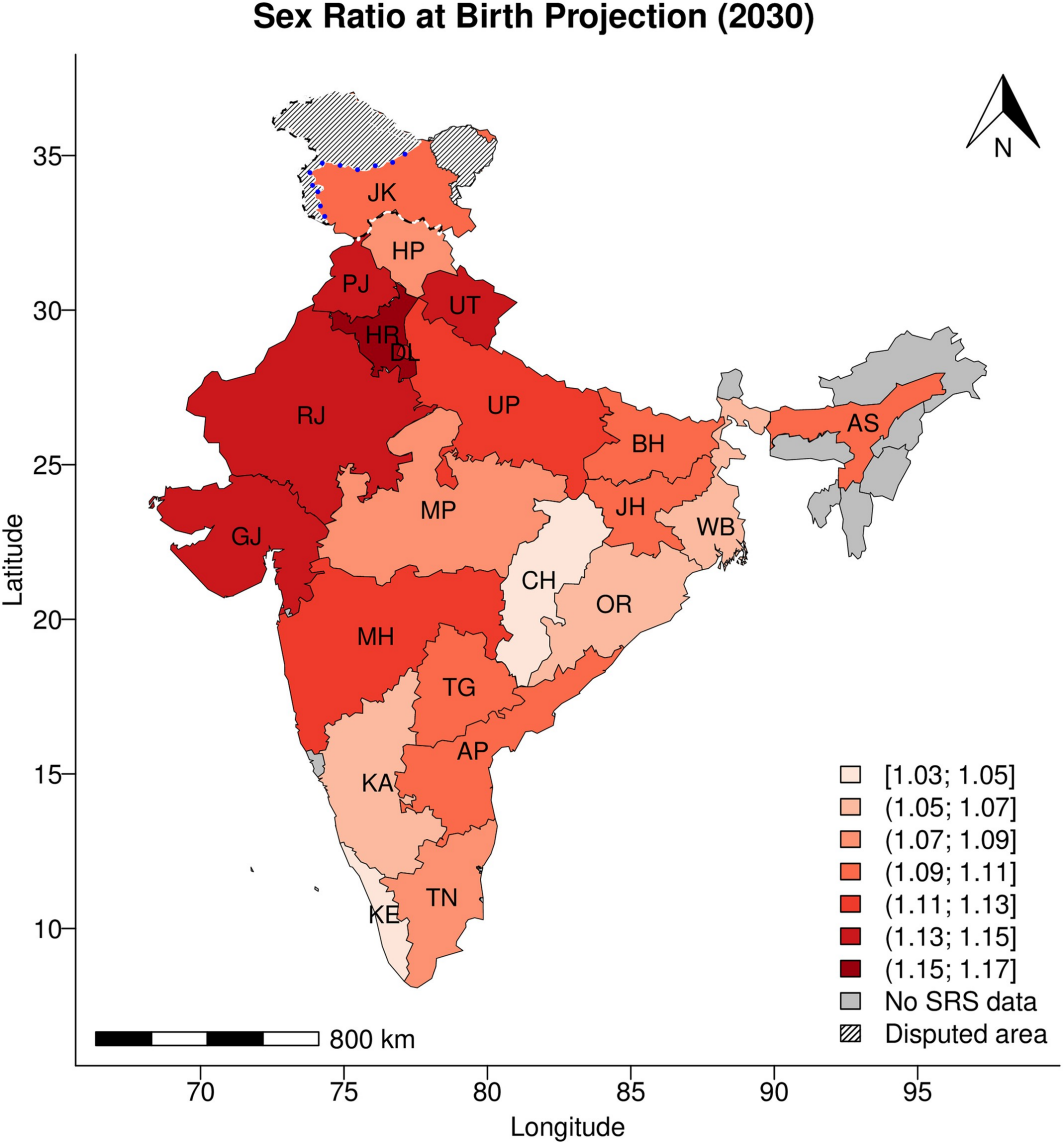
SRB median projections in 2030, by Indian State/UT. Results are shown for the 21 Indian States/UTs with SRS data. Values for Andhra Pradesh and Telangana are the same. State/UT names are: Andhra Pradesh (AP); Assam (AS); Bihar (BH); Chhattisgarh (CH); Delhi (DL); Gujarat (GJ); Haryana (HR); Himachal Pradesh (HP); Jammu and Kashmir (JK); Jharkhand (JH); Karnataka (KA); Kerala (KE); Madhya Pradesh (MP); Maharashtra (MH); Orissa (OR); Punjab (PJ); Rajasthan (RJ); Tamil Nadu (TN); Telangana (TG; same value as in AP); Uttar Pradesh (UP); Uttarakhand (UT); West Bengal (WB). The boundaries and names shown and the designations used on this map do not imply official endorsement. Blue dotted line represents approximately the Line of Control in Jammu and Kashmir agreed upon by India and Pakistan. The final status of Jammu and Kashmir has not yet been agreed upon by the parties.

Geographically, we project the SRB to vary greatly across the Indian States/UTs by 2030 (Fig 4). Generally speaking, the highest SRBs are concentrated in most of the northwestern States/UTs. The projected SRB becomes lower for the States/UTs that are further in the south, except for Chhattisgarh in central India. Chhattisgarh has one of the lowest SRBs during the projection period, but it is surrounded by states with much higher projected SRBs.

### State-specific case study

**Fig 5:**
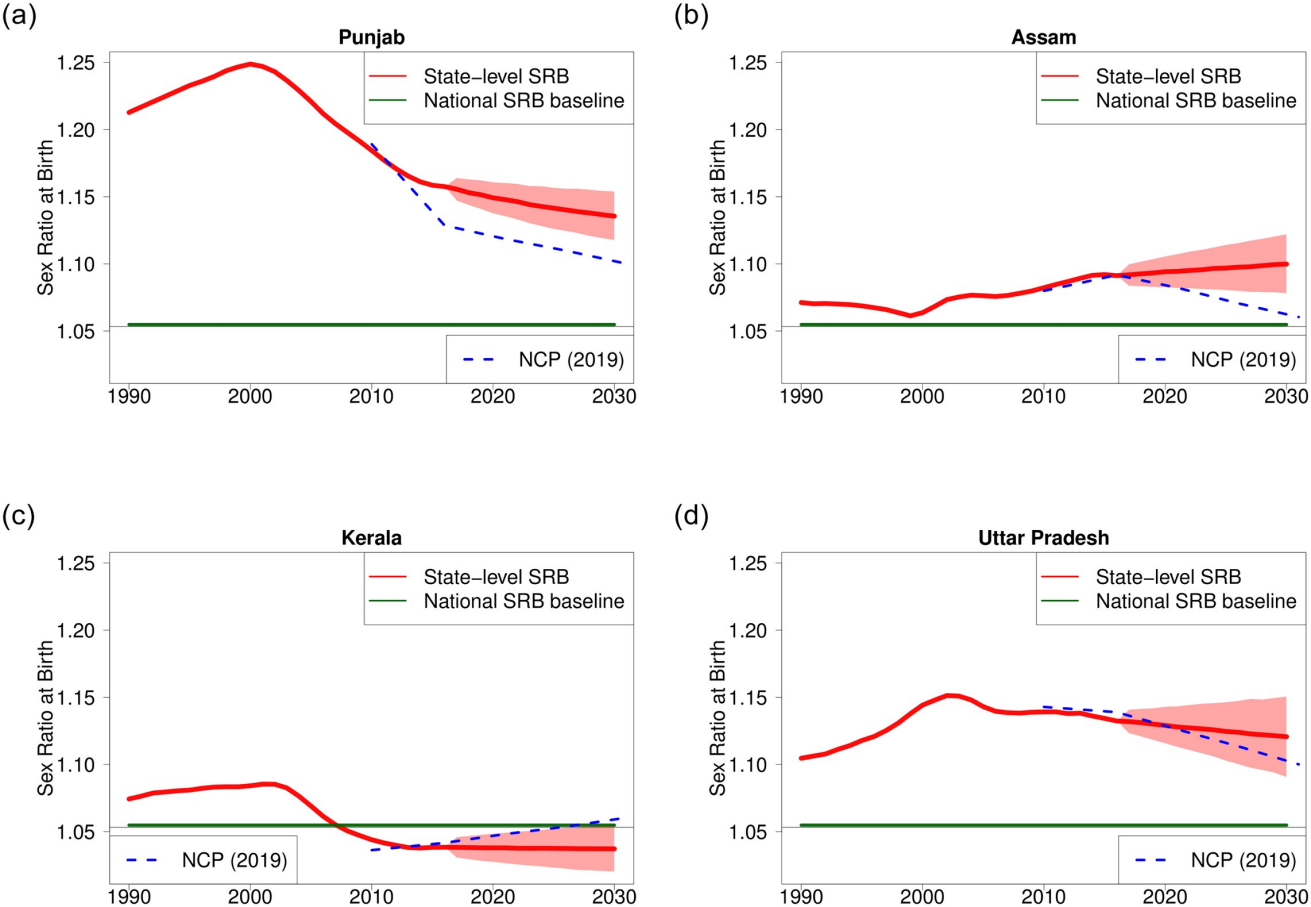
SRB projection for selected Indian states. The red line and shades are the median and 95% credible intervals of the state-specific SRB. The SRB median estimates before 2017 are from [8]. The green horizontal line refers to the SRB baseline for the whole India at 1.053 [13]. Blue dashed lines refer to projections from the National Commission on Population [15].

The SRB projections for four example Indian States/UTs shown in Fig 5 illustrate the broad diversity of SRB trajectories in India. The SRB projections from the NCP are shown in Fig 5 as comparison. We include the SRB projection comparison between our results and those from the NCP for 21 Indian States/UTs with SRS data in S1 Appendix.

The first case is that of Punjab, the region with one of the highest levels of gender bias. The SRB in this region was already above 1.200 in 1990, peaking at around 1.250 in the early 2000s. However, there has been a gradual decrease in SRB since then. Our model predicts that this decline will continue over the next decade. We project that the SRB in Punjab will decline steadily from 1.156 [1.147; 1.164] in 2017 to 1.136 [1.118; 1.154] by 2030. We found a similar pattern in the other northwestern states of Delhi and Haryana, where a rapid and real rise in the SRB was observed in the 1980s and 1990s. The SRB from NCP in 2016 is at 1.129, which reflects the level as suggested by the SRS data. Comparing to the NCP value, the SRB in Punjab in 2016 that we use in our study is estimated at 1.158 and is model-based and driven by both SRS and the 2015–2016 DHS data series [8]. The SRB from NCP is then linearly projected to 1.100 by 2031.

In Assam in northeast India, the SRB remained relatively normal until the late 1990s. However, the SRB started to climb steadily in the following years. Assam’s case is somewhat unique because it represents the only state, along with Andhra Pradesh, and Chhattisgarh, where our predictions point to a slight increase in SRB in the upcoming decade. The SRB in Assam is projected to continue to grow from 1.092 [1.084; 1.100] in 2017 to 1.100 [1.078; 1.122] in 2030, even though the progression is not statistically significant. In contract to the increasing trend that we project, the SRB from NCP is projected to decline and is linearly extrapolated from 1.092 in 2016 to 1.060 in 2031. The declining trend from NCP is because that the SRB level during 2015–2017 is below 1.100 and thus the SRB value in 2031 is assumed to be at 1.060.

Kerala has experienced historically low fertility rates dating as far back as the 1990s. Moreover, Kerala’s SRB had already declined below the national SRB baseline by the mid-2000s. We project the SRB in this state to remain around its current level of 1.038 [1.031; 1.046], reaching 1.037 [1.020; 1.055] by 2030. This SRB level will remain below 1.053, the SRB benchmark for the entire country. When looking at the projections by NCP, the SRB linearly increase from 1.042 in 2016 to 1.060 in 2031. Further research is required to verify if Kerala’s biological SRB is indeed lower than India’s or the low level of SRB is a temporal effect. It may be observed that the SRB in Sri Lanka–a country historically and geographically close to Kerala–has long oscillated between 1.03 and 1.05, according to birth registration statistics [13].

Uttar Pradesh is of primary importance since it is the most populous state in India with an estimated population of 237 million as of 2020. The SRB in Uttar Pradesh follows a similar rise and fall pattern; its SRB was above 1.100 in 1990 and reached the local maximum level of around 1.150 in the early 2000s. The SRB has declined slowly since then and it is projected to decrease further from 1.132 [1.123; 1.141] to 1.121 [1.092; 1.151] between 2017 and 2030. Gujarat and Rajasthan follow similar downward trends, which is of considerable importance to national sex imbalances at birth because these states contribute to almost half of the births for the entire country. While NCP also projected the SRB in Uttar Pradesh to decline, the trend is much steeper compare to ours; the SRB is linearly extrapolated from 1.139 in 2016 to 1.100 in 2031.

### Estimates of missing female births for Indian States/UTs

For the whole of India, summing up the 29 state-level projections, the cumulative number of missing female births (CMFB) during 2017–2030 is projected to be 6.8 [6.6; 7.0] million (Table 1). The average annual number of missing female births (AMFB) during 2017–2025 is projected to be 469 [456; 483] thousand. The average AMFB is projected to increase to 519 [485; 552] thousand during 2026–2030.

**Table 1:** Projection results for number of missing female births 2017–2030, for Indian States/UTs with imbalanced SRB.

India/Indian State/UT	AMFB (,000)		CMFB (,000)	Proportion of national CMFB (%)		
	2017–2025	2026–2030	2017–2030	2017–2025	2026–2030	2017–2030
India	469 [456; 483]	519 [485; 552]	6,820 [6, 613; 7, 023]	100	100	100
former state of Andhra Pradesh	29 [26; 31]	32 [27; 37]	418 [385; 452]	6.1 [5.6; 6.6]	6.2 [5.2; 7.2]	6.1 [5.6; 6.6]
Assam	11 [10; 12]	14 [11; 16]	166 [150; 183]	2.3 [2.1; 2.6]	2.6 [2.1; 3.2]	2.4 [2.2; 2.7]
Bihar	41 [35; 46]	44 [30; 58]	589 [503; 673]	8.7 [7.6; 9.8]	8.5 [6; 11]	8.6 [7.4; 9.8]
Delhi	7 [6; 7]	9 [8; 9]	105 [100; 110]	1.5 [1.4; 1.5]	1.7 [1.5; 1.9]	1.5 [1.4; 1.6]
Gujarat	38 [36; 41]	44 [39; 49]	565 [532; 599]	8.2 [7.6; 8.6]	8.5 [7.5; 9.6]	8.3 [7.8; 8.8]
Haryana	22 [21; 23]	24 [23; 26]	320 [309; 333]	4.7 [4.5; 4.9]	4.7 [4.3; 5.2]	4.7 [4.5; 4.9]
Himachal Pradesh	2 [2; 2]	2 [1; 2]	25 [22; 27]	0.4 [0.3; 0.4]	0.3 [0.3; 0.4]	0.4 [0.3; 0.4]
Jammu and Kashmir	4 [4; 4]	4 [4; 5]	59 [55; 63]	0.9 [0.8; 0.9]	0.9 [0.7; 1]	0.9 [0.8; 0.9]
Jharkhand	10 [9; 11]	11 [8; 14]	144 [127; 162]	2.1 [1.9; 2.4]	2.1 [1.6; 2.7]	2.1 [1.9; 2.4]
Madhya Pradesh	22 [19; 25]	23 [15; 30]	312 [262; 359]	4.7 [4; 5.4]	4.4 [3; 5.8]	4.6 [3.9; 5.3]
Maharashtra	49 [46; 52]	56 [49; 62]	722 [680; 763]	10.5 [9.8; 11.1]	10.8 [9.5; 12.1]	10.6 [10; 11.2]
Punjab	18 [17; 18]	18 [16; 20]	249 [238; 259]	3.8 [3.6; 3.9]	3.5 [3.1; 3.9]	3.6 [3.5; 3.8]
Rajasthan	49 [46; 52]	56 [49; 64]	722 [677; 767]	10.4 [9.8; 11.1]	10.9 [9.5; 12.3]	10.6 [9.9; 11.3]
Tamil Nadu	14 [12; 16]	15 [11; 19]	199 [175; 225]	3 [2.6; 3.4]	2.9 [2.1; 3.6]	2.9 [2.6; 3.3]
Uttar Pradesh	141 [131; 150]	151 [125; 176]	2,020 [1, 865; 2, 173]	30 [28.4; 31.5]	29.1 [25.2; 32.6]	29.6 [27.9; 31.3]
Uttarakhand	6 [6; 6]	6 [6; 7]	87 [83; 90]	1.3 [1.2; 1.4]	1.2 [1.1; 1.4]	1.3 [1.2; 1.3]

Median projection and 95% credible intervals for (i) the average of annual number of missing female births (AMFB) in thousands; (ii) the cumulative number of missing female births (CMFB) in thousands; (ii) the proportion of state-level CMFB to the national (sum of all 29 States/UTs) CMFB; for periods 2017–2025, 2026–2030, and 2017–2030, by Indian State/UT. Numbers in brackets are 95% credible intervals. State-level proportions do not sum up to 100% because results from 16 States/UTs with imbalanced SRB and with SRS data are shown. States/UTs are ordered alphabetically.

Among all States/UTs, Uttar Pradesh has the largest contribution to the number of missing female births: its CMFB for 2017–2030 is projected to be 2.0 [1.9; 2.2] million, representing 29.6% [27.9%; 31.3%] of the national total. Over the entire projection period from 2017 to 2030, its share of the total national CMFB is projected to remain close to 30%. The average AMFB in Uttar Pradesh is projected to be 141 [131; 150] thousand during 2017–2025 and increase to 151 [125; 176] thousand during 2026–2030.

### Validation results

The validation results indicate reasonably good calibrations and predicting power of the model. Table 2 summarizes the results related to the left-out SRB median estimates from prior study [8] for the out-of-sample validation exercise. Median errors and median absolute errors are very close to zero. The coverage of 95% and 80% prediction intervals are around the expected values. Table 3 shows results for the comparison between model estimates obtained based on the full dataset and based on the training set for the out-of-sample validation exercise. Median errors and the median absolute errors are close to zero. The proportions of updated estimates that fall below the credible intervals constructed based on the training set are reasonable, with at most four state-level estimates falling outside their respective bounds.

**Table 2:** Validation results for left-out SRB observations.

# States/UTs in test dataset	29
Median error	0.001
Median absolute error	0.002
Below 95% prediction interval (%)	0.0
Above 95% prediction interval (%)	3.4
**Expected (%)**	**2.5**
Below 80% prediction interval (%)	3.4
Above 80% prediction interval (%)	9.5
**Expected (%)**	**10**

Error is defined as the difference between a left-out SRB median estimate from prior study [8] and the posterior median of its predictive distribution.

**Table 3:** Validation results for estimates based on training set, by left-out year.

Out-of-Sample Validation	2012	2013	2014	2015	2016
Median error	0.000	0.001	0.001	0.003	0.003
Median absolute error	0.000	0.001	0.002	0.003	0.003
Below 95% credible interval (%)	0.0	0.0	0	0.0	0.0
Above 95% credible interval (%)	0.0	3.4 (1)	3.4 (1)	3.4 (1)	3.4 (1)
**Expected (%)**	≤**2.5**	≤**2.5**	≤**2.5**	≤**2.5**	≤**2.5**
Below 80% credible interval (%)	0.0	3.4 (1)	3.4 (1)	3.4 (1)	3.4 (1)
Above 80% credible interval (%)	0.0	3.4 (1)	10.3 (3)	13.8 (4)	10.3 (3)
**Expected (%)**	≤**10**	≤**10**	≤**10**	≤**10**	≤**10**

Error is defined as the differences between a model estimate for SRB based on full dataset and training set. The proportions refer to the proportions (%) of Indian States/UTs in which the median estimates based on the full dataset fall below or above their respective 95% and 80% credible intervals based on the training set. Numbers in the parentheses after the proportions indicate the number of countries in a certain year where the median estimates based on the full dataset fall below or above their respective 95% and 80% credible intervals based on the training set.

## Discussion

This work represents the first study to provide projections of SRB at the state level in India, including measurements of uncertainty based on reproducible models. In this projection model, we take into account two essential factors, the DSRB and the TFR, that lead to sex-selective abortion and consequently skew the SRB. This is achieved by producing DSRB projection based on a Bayesian hierarchical model, and by making use of the existing projections of TFR from other studies [16]. We project that out of the 21 Indian States and UTs with SRS data, 16 will have imbalanced SRB between 2017 and 2030. Among these 16 States/UTs, the largest contribution to the female births deficit is projected to be from Uttar Pradesh, with a cumulative number of missing female births projected to be 2.0 [1.9; 2.2] million from 2017 to 2030. The total female birth deficit during 2017–2030 for the whole of India is projected to be 6.8 [6.6; 7.0] million.

Our SRB projections represent an essential input for the population projection models in India, especially at the sub-national level. The NCP projections of SRB uses simple linear extrapolation and assumes the state-level SRB in 2031 is either 1.100 (if SRB during 2015–2017 is above 1.100) or 1.060 (if SRB during 2015–2017 is below 1.100) [15]. In our model, however, we assume the non-linear nature of the sex ratio transition and allows for the SRB imbalance process differ in levels and trends across States/UTs due to India’s unique social and demographic diversity. Recent projections of India’s population by age, sex, and educational attainment and by state and type of residence to 2100 [16], assume that all SRBs will monotonically convergence to a certain value in the future. However, long-term population projections are sensitive to the projected SRB, especially in India. Hence, our probabilistic projection of SRB will contribute to more precise simulations of the long-term impact of SRB and the uncertainty it imposes on various population indicators.

The choice of regression predictors in our model depends not only on how well the regression predictors can approximate the effects of son preference and fertility squeeze, but also on the reliability and availability of their projections. When interpreting the projected SRB, it is worthwhile to note that the results are based on the model assumptions derived using a set of predictors selected for the projection model. Although the sex ratio for the last birth (SRLB) is a more stable indicator than DSRB for measuring son preference, we chose to use DSRB because the SRLB data are subject to relatively large sampling errors. Thus, we opted for the DSRB as an indicator for son preference as it has clear trends over time. Note also that we did not incorporate the predictors which represent technology diffusion in the projection model due to lack of adequate measurements and data collections. There are state-level variables such as the proportion of women availing of ultrasounds during their most recent birth, the proportion of women delivering in health institutions, or the share of the private health system. Nonetheless, the quality of these state-specific data may not be as good as the quality of birth-related information. Consequently, it is challenging to produce reliable projections for indicators that could be used as a proxy for technology diffusion for each Indian State/UT.

Our Bayesian probabilistic projections of SRB and missing female births by Indian State/UT underscore the importance of monitoring the SRB over time at the sub-national level, especially in countries such as India that experience severe ongoing SRB imbalance in a highly heterogeneous demographic context. In this way, even with limited healthcare resources, the future abortion of girls in favor of male offspring can be minimized through better identification, monitoring, and education in the worst affected regions. In view of the large contribution of India to the global number of missing female births, interventions towards a reduction of son preference and sex-selective behavior by Indian couples remain key to a gradual normalization of the sex ratio at birth in the world. Our study highlights the need to strengthen policies that advocate for gender equity and the introduction of support measures to counteract existing gender biases that adequately target each regional context. Future work may include additional sources of heterogeneity, such as education, religion, and ethnicity, for projecting the SRB in India and extending the SRB predictions for longer-term projections.

## Supporting information

S1 Appendix Data preprocessing, model specifications, computing details, and additional figures and tables. (PDF)

S1 Table Indian States/UTs classification based on data quality and SRB imbalances. The red numbers at the beginning of each cell refer to the number of States/UTs that fall under each category.
(PDF)

## References

[1] Attané I, Guilmoto CZ. Watering the neighbour’s garden: The growing demographic female deficit in Asia. Paris: Committee for International Cooperation in National Research in Demography; 2007.

[2] Bongaarts J, Guilmoto CZ. How many more missing women? Excess female mortality and prenatal sex selection, 1970–2050. Population and Development Review. 2015;41(2):241–269. 10.1111/j.1728-4457.2015.00046.x

[3] Das Gupta M, Zhenghua J, Bohua L, Zhenming X, Chung W, Hwa-Ok B. Why is son preference so persistent in East and South Asia? A cross-country study of China, India and the Republic of Korea. The Journal of Development Studies. 2003;40(2):153–187. 10.1080/00220380412331293807

[4] Guilmoto CZ. Sex Imbalances at Birth: Current trends, consequences and policy implications. Bangkok, Thailand: UNFPA Asia and Pacific Regional Office; 2012. Available from: http://www.unfpa.org/webdav/site/global/shared/documents/publications/2012/SexImbalancesatBirth.PDFUNFPAAPROpublication2012.pdf.

[5] Guilmoto CZ. The sex ratio transition in Asia. Population and Development Review. 2009;35(3):519–549. 10.1111/j.1728-4457.2009.00295.x

[6] Guilmoto CZ. Skewed sex ratios at birth and future marriage squeeze in China and India, 2005–2100. Demography. 2012;49(1):77–100. 10.1007/s13524-011-0083-7 22180130

[7] Jha P, Kumar R, Vasa P, Dhingra N, Thiruchelvam D, Moineddin R. Low male-to-female sex ratio of children born in India: national survey of 1⋅1 million households. The Lancet. 2006;367(9506):211–218. 10.1016/S0140-6736(06)67930-0 16427489

[8] Chao F, Yadav AK. Levels and trends in the sex ratio at birth and missing female births for 29 states and union territories in India 1990–2016: A Bayesian modeling study. Foundations of Data Science. 2019;1(2):177–196. 10.3934/fods.2019008

[9] Jha P, Kesler MA, Kumar R, Ram F, Ram U, Aleksandrowicz L, et al Trends in selective abortions of girls in India: analysis of nationally representative birth histories from 1990 to 2005 and census data from 1991 to 2011. The Lancet. 2011;377(9781):1921–1928. 10.1016/S0140-6736(11)60649-1 PMC3166246 21612820

[10] Ravinder Kaur, Surjit S Bhalla, Manoj K Agarwal Prasanthi Ramakrishnan. Sex Ratio at Birth—The Role of Gender, Class and Education. United Nations Population Fund India; 2017.

[11] Roy TK, Chattopadhyay A. Daughter discrimination and future sex ratio at birth in India. Asian Population Studies. 2012;8(3):281–299. 10.1080/17441730.2012.714669

[12] United Nations, Department of Economic and Social Affairs, Population Division. World Population Prospects: The 2019 Revision; 2019.

[13] Chao F, Gerland P, Cook AR, Alkema L. Systematic assessment of the sex ratio at birth for all countries and estimation of national imbalances and regional reference levels. Proceedings of the National Academy of Sciences. 2019;116(19):9303–9311. 10.1073/pnas.1812593116 PMC6511063 30988199

[14] Kashyap R, Villavicencio F. The dynamics of son preference, technology diffusion, and fertility decline underlying distorted sex ratios at birth: A simulation approach. Demography. 2016;53(5):1261–1281. 10.1007/s13524-016-0500-z 27638765 PMC5566175

[15] National Commission on Population, Ministry of Health and Family Welfare. Population Projections for India and States 2011-2036, Report of the Technical Group on Population Projections; 2019.

[16] KC S, Wurzer M, Speringer M, Lutz W. Future population and human capital in heterogeneous India. Proceedings of the National Academy of Sciences. 2018;115(33):8328–8333. 10.1073/pnas.1722359115 PMC6099904 30061391

[17] Bongaarts J. The implementation of preferences for male offspring. Population and Development Review. 2013;39(2):185–208. 10.1111/j.1728-4457.2013.00588.x

[18] Yue YR, Simpson D, Lindgren F, Rue H, et al Bayesian adaptive smoothing splines using stochastic differential equations. Bayesian Analysis. 2014;9(2):397–424. 10.1214/13-BA866

[19] Alkema L, Raftery AE, Gerland P, Clark SJ, Pelletier F, Buettner T, et al Probabilistic projections of the total fertility rate for all countries. Demography. 2011;48(3):815–839. 10.1007/s13524-011-0040-5 21748544 PMC3367999

[20] Simpson D, Rue H, Riebler A, Martins TG, Sørbye SH, et al Penalising model component complexity: A principled, practical approach to constructing priors. Statistical Science. 2017;32(1):1–28. 10.1214/16-STS576

[21] International Institute for Population Sciences (IIPS) and ICF. National Family Health Survey (NFHS-4), 2015-16: India; 2017.

[22] Guilmoto CZ, Chao F, Kulkarni PM. On the estimation of female births missing due to prenatal sex selection. Population Studies. 2020;74(2):283–289. 10.1080/00324728.2020.1762912 32489140

