# Supplementary material for: Probabilistic projection of the sex ratio at birth and missing female births by State and Union Territory in India
Source: PLoS One. 2020 Aug 19;15(8):e0236673. doi: 10.1371/journal.pone.0236673 (PMC7446920; doi:10.1371/journal.pone.0236673)
Supplement: S1 Appendix — (PDF) [file pone.0236673.s001.pdf]

S1 Appendix
Probabilistic projection of the sex ratio at birth and missing female births by State and Union Territory in India

Fengqing Chao*[1], Christophe Z. Guilmoto[2], Samir K.C.[3,4], and Hernando Ombao[1]

[1]Statistics Program, Computer, Electrical and Mathematical Sciences and Engineering Division, King Abdullah University of Science and Technology (KAUST), Thuwal, Saudi Arabia

[2]CEPED/IRD, Université de Paris, Paris, France

[3]Asian Demographic Research Institute, Shanghai University, Shanghai, China

[4]Wittgenstein Centre for Demography and Global Human Capital (Univ.Vienna, IIASA, VID/OeAW), International Institute for Applied Systems Analysis, Laxenburg, Austria

July 19, 2020

*Corresponding author (FC); Email: fengqing.chao@kaust.edu.sa

Contents

1 Data Pre-processing **1**
1.1 Sampling Errors for State-level Desired Sex Ratio at Birth Observations 1
1.2 State-level TFR Data 1

2 Model for State-level Desired Sex Ratio at Birth **1**

3 Statistical Computing **2**

4 Estimates of sex-specific live births, missing female births **2**

5 Model Summary **3**

6 Supplementary Figures **5**

List of Tables

1 Notation summary 4

List of Figures

1 Desired sex ratio at birth by Indian states, 1990–2040 6
2 Total fertility rate by Indian states, 1990–2030 7
3 Number of births by Indian states, 2017–2030 8
4 SRB estimates and projections by Indian states, 1990–2030 8

List of Abbreviations

AMFB	Annual number of Missing Female Births
AR(1)	Auto-Regressive model of order 1
CMFB	Cumulative number of Missing Female Births
DHS	Demographic and Health Survey
DSRB	Desired Sex Ratio at Birth
MCMC	Markov chain Monte Carlo
RW2	second-order Random Walk
PC	Penalized Complexity
SRB	Sex Ratio at Birth
SRLB	Sex Ratio at Last Birth
SRS	Sample Registration System
TFR	Total Fertility Rate
UT	Union Territory

1 Data Pre-processing

1.1 Sampling Errors for State-level Desired Sex Ratio at Birth Observations

We process the individual-level data and household data from the four India DHS 1992–1993, 1998–1999, 2005–2006 and 2015–2016 to compute the observations and corresponding sampling errors of desired sex ratio at birth (DSRB). India DHS, also known as the National Family Health Survey (NFHS), adopt a two-stage stratified sampling frame for rural areas and a three-stage sample design for urban areas, except for the 2015–2016 DHS where two-stage sampling frame were applied to both rural and urban areas [8]. The first stage is to sample the primary sampling units with probability proportional to population size, followed by random selection of census enumeration block and/or households. To simplify, all notations in this section refer to state level in India, for a specific India DHS survey. Hence, we remove the subscription c from all notations in this section.

For a specific DHS survey, we calculate the Jackknife sampling error for log-transformed DSRB at the time when women were interviewed. The reference year t of the DSRB for a DHS survey is taken as the mid point of the survey fieldwork period. Let U denote the total number of clusters or primary sampling units. The u-th partial prediction of DSRB is given by:

$$d_{-u} = \frac{\sum_{m=1}^{M} \mathbb{I}_m(v_m \neq u) B_m w_m}{\sum_{m=1}^{M} \mathbb{I}_m(v_m \neq u) G_m w_m}, \text{ for } u \in \{1, \ldots, U\},$$

where m indexes each women interviewed in a DHS survey during the reproductive age under 35 years old, and M is the total number of such women in a survey. v_m is the cluster number of the m-th woman, B_m and G_m are the desired number of boys and girls[1] respectively for the m-th women, w_m is the sampling weight for the m-th woman. $\mathbb{I}(\cdot) = 1$ if the condition inside brackets is true and $\mathbb{I}(\cdot) = 0$ otherwise. The u-th pseudo-value estimate of DSRB on the log-scale is:

$$\log(d)_u^* = U \log(d_{obs}) - (U-1)\log(d_{-u}),$$

where $d_{obs} = \frac{\sum_{m=1}^{M} B_m w_m}{\sum_{n=1}^{N} G_m w_m}$.

The Jackknife variance of log-transformed DSRB is:

$${\sigma_D}^2 = \frac{\sum_{u=1}^{U}\left(\log(d)_u^* - \overline{\log(d)}\right)^2}{U(U-1)}.$$

where $\overline{\log(d)} = \frac{1}{U}\sum_{u=1}^{U}\log(d)_u^*$.

1.2 State-level TFR Data

TFR data by Indian State/UT during 1990–2016 are primarily from the India Sample Registration System (SRS). The TFR values in Kerala in 1991 and 1994 are taken from [10]. The TFR projections by Indian State/UT during 2017–2030 are from [9].

2 Model for State-level Desired Sex Ratio at Birth

For the i-th observation of the log of desired sex ratio at birth (DSRB) d_i, we assume it follows a normal distribution on the log-scale:

$$d_i \sim \mathcal{N}(D_{c[i],t[i]}, {\sigma_D}_i^2 + \omega^2), \text{ for } i \in \{1, \cdots, 101\}. \quad (1)$$

[1] If a woman have no preference of boys or girls, we assume that $B_m = G_m = T_m/2$, where T_m is the ideal number of children for the m-th woman.

The mean of the distribution $D_{c[i],t[i]}$ is the true DSRB on the log-scale for state $c[i]$ in year $t[i]$ for the i-th observation. The model of the mean is explained in the rest of this section. The variance of the distribution is the sum of sampling and non-sampling variances. $\sigma_{D_i^2}$ is the sampling variance for the i-th observation computed using the Jackknife method (see Section 1.1). ω^2 is the non-sampling variance parameter for DHS survey data (hence estimated in the model), representing the data errors that are not possible to quantify or be eliminated mainly due to non-response, recall errors, and data recording errors.

3 Statistical Computing

Computing of SRB Model We use the `R`-package **INLA** [16] for model fitting of the state-level SRB.

Computing of DSRB Model We obtained posterior samples of all the model parameters and hyper parameters using a Markov chain Monte Carlo (MCMC) algorithm, implemented in the open source softwares `R 3.6.1` [15] and `JAGS 4.3.0` [14] (Just another Gibbs Sampler), using `R`-packages **R2jags** [19] and **rjags** [12]. Results were obtained from 8 chains with a total number of 1,000 iterations in each chain, while the first 2,000 iterations were discarded as burn-in. After discarding burn-in iterations and proper thinning, the final posterior sample size for each parameter is 8,000. Convergence of the MCMC algorithm and the sufficiency of the number of samples obtained were checked through visual inspection of trace plots and convergence diagnostics of Gelman and Rubin [5], implemented in the **coda** `R`-package [13].

4 Estimates of sex-specific live births, missing female births

To quantify the effect of SRB imbalance due to sex-selective abortion, we calculate the annual number of missing female births (AMFB) and the cumulative number of missing female births (CMFB) over time. The estimated and expected female live births for an Indian State/UT c in year t, denoted as $B_{c,t}^F$ and $B_{c,t}^{FE}$ respectively, are obtained as follows [6]:

$$\begin{aligned} B_{c,t}^F &= \tfrac{B_{c,t}}{1+R_{c,t}}, \\ B_{c,t}^{FE} &= \tfrac{B_{c,t}-B_{c,t}^F}{N}, \end{aligned}$$

where N is the SRB baseline for the whole of India [2, 3]. $B_{c,t}$ is the total number of births for Indian State/UT c year t [9], as illustrated in Figure 3.

The annual number of missing female births (AMFB) for an Indian State/UT c in year t is defined as:

$$B_{c,t}^{F*} = B_{c,t}^{FE} - B_{c,t}^F.$$

The cumulative number of missing female births (CMFB) for a period t_1 to t_2 in an Indian State/UT c is defined as the sum of AMFBs from the year t_1 up to the year t_2:

$$Z_{c,[t_1,t_2]}^{F*} = \sum_{t=t_1}^{t_2} B_{c,t}^{F*}.$$

An Indian State/UT is identified to have an SRB imbalance if its AMFB in at least one year since 2017 is above zero for more than 95% of the posteriors samples:

$$\sum_{t=2017}^{2030} \mathbb{I}_t \left\{ \frac{1}{G} \sum_{g=1}^{G} \mathbb{I}_g \left[\left(B_{c,t}^{F*}\right)^{(g)} > 0 \right] > 95\% \right\} \geq 1,$$

where $\left(B_{c,t}^{F*}\right)^{(g)}$ is the g-th posterior sample of the AMFB for Indian State/UT c in year t.

5 Model Summary

Table 1 summarizes the notations and indexes used in this paper.

Model for State-level Sex Ratio at Birth

$$
\begin{aligned}
s_i &\sim \mathcal{N}(S_{c[i],t[i]}, 0.001^2), \text{ for } i \in \{1, \cdots, 566\}, \\
S_{c,t} &= \log(N) + P_{c,t}, \text{ for } \forall c, \forall t, \\
V_{c,t} &= \alpha_c D_{c,t+5} + f_c\left(F_{c,t}\right), \text{ for } \forall c, \forall t, \\
P_{c,t} | V_{c,t} &\sim \mathcal{N}(V_{c,t}, \sigma_\epsilon^2 / (1 - \rho_c^2)), \text{ for } \forall c, t = 1, \\
P_{c,t} | P_{c,t-1}, V_{c,t} &= V_{c,t} + \rho_c (P_{c,t-1} - V_{c,t}) + \epsilon_{c,t}, \text{ for } \forall c, t \in \{2, \cdots, T\}, \\
\alpha_c | \tau_\alpha &\overset{\text{i.i.d.}}{\sim} \mathcal{N}(0, \tau_\alpha^{-1}), \text{ for } \forall c, \\
\epsilon_{c,t} &\sim \mathcal{N}(0, \tau_{\epsilon_c}^{-1}), \text{ for } \forall c, t \in \{2, \cdots, T\}, \\
f_c\left(F_{c,t}\right) = \Delta_{c,t}^2 &= F_{c,t} - 2F_{c,t-1} + F_{c,t-2}, \text{ for } \forall c, t \in \{3, \cdots, T\}, \\
\Delta_{c,t}^2 &\sim \mathcal{N}(0, \tau_c^{-1}), \text{ for } \forall c, t \in \{3, \cdots, T\}.
\end{aligned}
$$

Priors for State-level Sex Ratio at Birth Model The state-specific auto-regressive parameter ρ_c and τ_{ϵ_c} and the log-precision parameter $\log(\tau_\alpha)$ for the state-specific DSRB coefficient are assigned with Penalized Complexity (denoted as $\mathcal{PC}$) priors as explained in [17].

$$
\begin{aligned}
\log\left(\frac{\tau_c}{\eta_c}\right) &\sim \mathcal{PC}_{\text{prec}}(u = \nu, \alpha = 0.01), \text{ for } \forall c, \\
\rho_c &\sim \mathcal{PC}_{\text{cor1}}(u = 0.8, \alpha = 0.5), \text{ for } \forall c, \\
\phi_c = \log(\tau_{\epsilon_c}) &\sim \mathcal{PC}_{\text{prec}}(u = 1, \alpha = 0.01), \text{ for } \forall c, \\
\log(\tau_\alpha) &\sim \mathcal{PC}_{\text{prec}}(u = \nu, \alpha = 0.01), \text{ for } \forall c.
\end{aligned}
$$

where $\nu = 0.042$ is the standard deviation of all the observations on the log-scale.

The density for ρ_c prior $\mathcal{PC}_{\text{cor1}}(u, \alpha)$ is:

$$
\begin{aligned}
\pi(\rho_c) &= \frac{\lambda \exp\left\{-\lambda\sqrt{1-\rho_c}\right\}}{1 - \exp\left\{-\sqrt{2}\lambda\right\}} \frac{1}{2\sqrt{1-\rho_c}}, \\
\frac{\exp\left\{-\lambda\sqrt{1-u}\right\}}{1 - \exp\left\{-\sqrt{2}\lambda\right\}} &= \alpha.
\end{aligned}
$$

The density for the log-precision $\phi_c = \log(\tau_{\epsilon_c})$ prior $\mathcal{PC}_{\text{prec}}(u, \alpha)$ is:

$$
\begin{aligned}
\pi(\phi_c) &= \frac{\lambda}{2} \exp\left\{-\lambda \exp\left\{-\frac{\phi_c}{2}\right\} - \frac{\phi_c}{2}\right\}, \\
\lambda &= \frac{\log(\alpha)}{u}.
\end{aligned}
$$

The log precision $\log(\tau_c)$ is scaled such that $f_c\left(F_{c,t}\right)$ has a generalized variance equal to 1 [18]. η_c is a value where INLA auto-generated.

State-level SRB model	
**Symbol**	**Description**
i	Indicator for the i-th SRB estimate during 1990–2016 for model input across all state-years, $i \in \{1, \ldots, 566\}$.
t	Indicator for year, $t \in \{1, \ldots, T\}$. $t = 1$ refers to year 1990 and $t = T$ refers to year 2030.
c	Indicator for Indian State/UT, $c \in \{1, \ldots, C\}$, where $C = 29$.
s_i	The i-th SRB estimate on the log-scale during 1990–2016 for model input, taken from [4].
$R_{c,t}$	The model fitting for the true SRB for State/UT c in year t.
$S_{c,t}$	The model fitting for the true SRB on the log-scale for State/UT c in year t. $S_{c,t} = \log(R_{c,t})$.
N	The baseline level of SRB for the whole India. $N = 1.053$ [2, 3].
$P_{c,t}$	The difference between $S_{c,t}$ and $\log(N)$ for State/UT c in year t. $P_{c,t} = S_{c,t} - \log(N)$
$V_{c,t}$	The conditional mean for $P_{c,t}$.
$D_{c,t+5}$	The log of desired sex ratio at birth (DSRB) for state c in year $t + 5$. $D_{c,t+5}$ is used to correspond to $V_{c,t}$, where the 5-year time lag between $D_{c,t+5}$ and $V_{c,t}$ is to reflect the assumption that the DSRB generated from DHS of women under age 35 should represent the desire at the time before the first births [1].
$F_{c,t}$	The log of total fertility rate (TFR).
$f_c(F_{c,t})$	The state-specific non-linear function with RW2 structure for $F_{c,t}$.
ρ_c	State-specific autoregressive parameter in AR(1) time series model for $P_{c,t}$.
τ_{ϵ_c}	State-specific precision of distortion parameter in AR(1) time series model for $P_{c,t}$.
α_c	The state-specific coefficient parameter for $D_{c,t+5}$.
**State-level DSRB model**	
**Symbol**	**Description**
i	Indicator for the i-th DSRB observation across all state-years, $i \in \{1, \ldots, 101\}$.
t	Indicator for year, $t = 1, \ldots, T$. $t = 1$ refers to year 1990 and $t = T$ refers to year 2035.
c	Indicator for Indian State/UT, $c \in \{1, \ldots, C\}$, where $C = 29$.
d_i	The i-th DSRB observation on log-scale across all state-years.
σ_{Di}	The i-th sampling error for log-scaled DSRB observation, which is a pre-calculated value.
$D_{c,t}$	The model fitting for the true DSRB on log-scale for State/UT c in year t.
$\Delta_{c,t}$	The difference between $\exp\{D_{c,t}\}$ and 1 for State/UT c in year t. $\Delta_{c,t} = \exp\{D_{c,t}\} - 1$.
ϕ_c	The state-specific coefficient parameter of the logit function of $\Delta_{c,t}$.
ζ_c	The state-specific intercept parameter of the logit function of $\Delta_{c,t}$.
δ_c	The state-specific scale parameter of the logit function of $\Delta_{c,t}$.
μ_ϕ and σ_ϕ	The global mean and standard error parameters for ϕ_c.
μ_ζ and σ_ζ	The global mean and standard error parameters for ζ_c.
μ_δ and σ_δ	The global mean and standard error parameters for δ_c.
ω	The non-sampling error parameter for every log-scaled DSRB observation d_i.

Table 1: **Notation summary.**

Model for State-level Desired Sex Ratio at Birth

$$
\begin{aligned}
d_i &\sim \mathcal{N}(D_{c[i],t[i]}, \sigma_{D_i}^2 + \omega^2), \text{ for } i \in \{1, \cdots, 101\}, \\
\exp\{D_{c,t}\} &= 1 + \Delta_{c,t}, \text{ for } \forall c, \forall t, \\
\Delta_{c,t} &= \frac{\delta_c \cdot \exp\{\phi_c \cdot \log(t) + \zeta_c\}}{1 + \exp\{\phi_c \cdot \log(t) + \zeta_c\}}, \text{ for } \forall c, \forall t, \\
\delta_c &\sim \mathcal{N}(\mu_\delta, \sigma_\delta^2), \text{ for } \forall c, \\
\phi_c &\sim \mathcal{N}(\mu_\phi, \sigma_\phi^2), \text{ for } \forall c, \\
\zeta_c &\sim \mathcal{N}(\mu_\zeta, \sigma_\zeta^2), \text{ for } \forall c, \\
\mu_\delta &\sim \mathcal{U}(-0.5, 0.5), \\
\mu_\phi &\sim \mathcal{U}(-0.5, 0.5), \\
\mu_\zeta &\sim \mathcal{U}(-0.5, 0.5), \\
\sigma_\delta &\sim \mathcal{U}(0, 2), \\
\sigma_\phi &\sim \mathcal{U}(0, 2), \\
\sigma_\zeta &\sim \mathcal{U}(0, 2), \\
\omega &\sim \mathcal{U}(0.05, 2).
\end{aligned}
$$

$\mathcal{U}(a, b)$ denotes a continuous uniform distribution with lower and upper bounds at a and b respectively.

6 Supplementary Figures

The covariates and other data used for the projection are illustrated in the following plots:

- Figure 1: Desired sex ratio at birth by Indian State/UT, 1990–2040, used as a covariate in the model.
- Figure 2: Total fertility rate by Indian State/UT, 1990–2030 [9], used as a covariate in the model.
- Figure 3: Number of births by Indian State/UT, 2017–2030 [9], used to compute number of missing female births by Indian State/UT.
- Figure 4: SRB estimates and projections by Indian State/UT, 1990–2030.

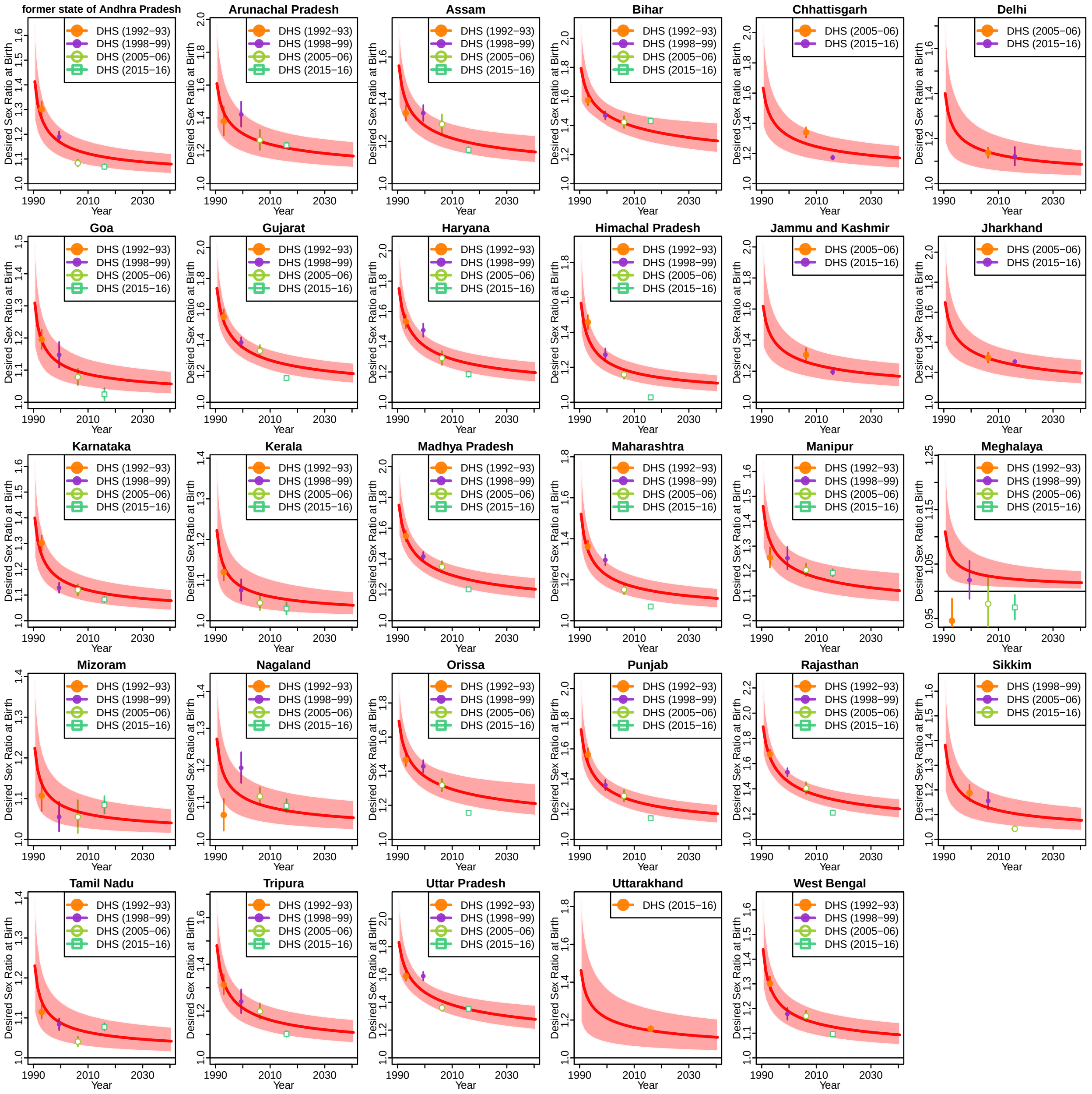

Figure 1: **Desired sex ratio at birth (DSRB) by Indian states, 1990–2040.** The red line and shades are the median and 95% credible intervals of the state-specific DSRB. Data from different surveys are differentiated by dot shapes and colors. Vertical line segments around the data represent the sampling variability in the observations (quantified by two times the /sampling standard errors).

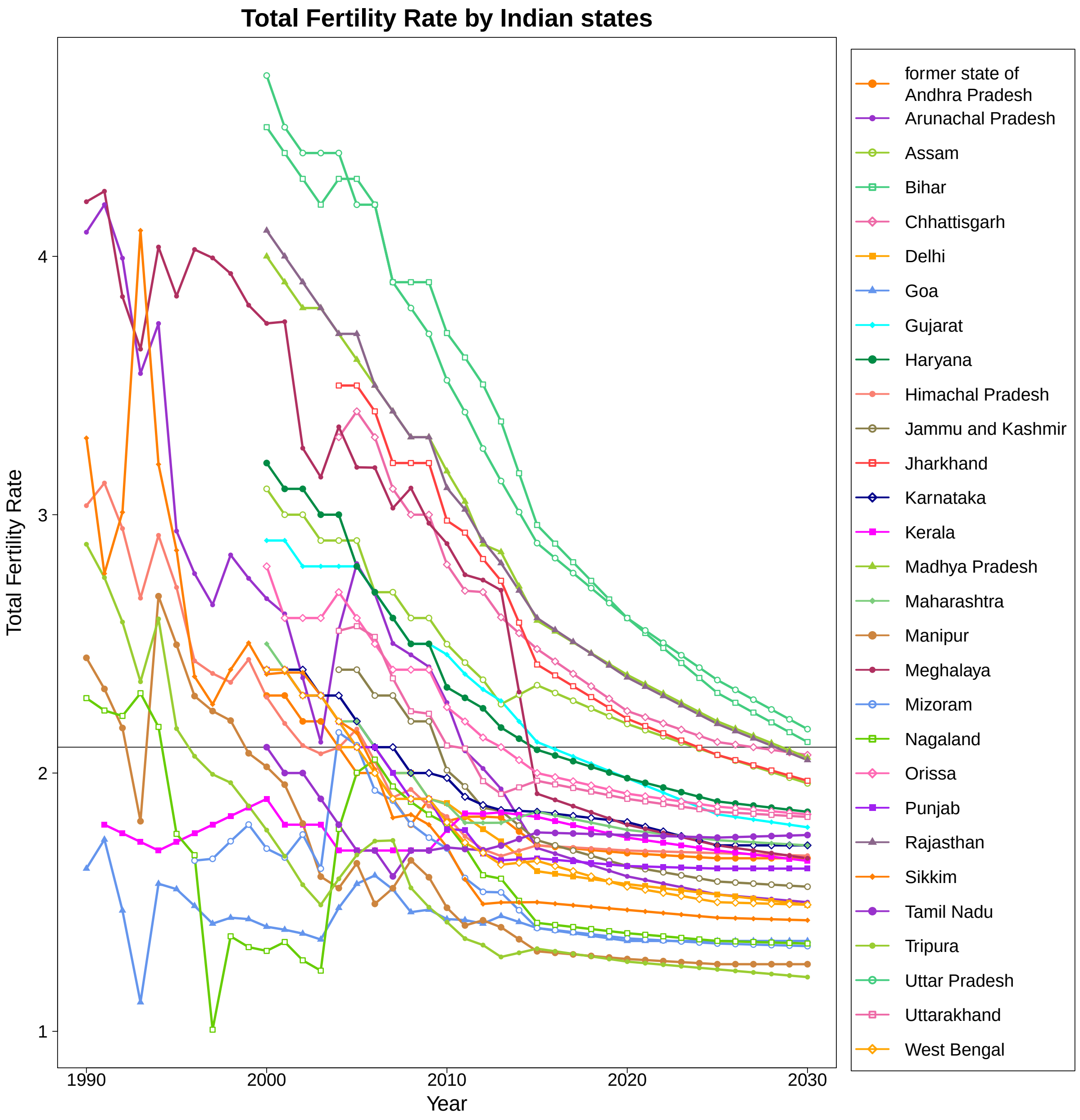

Figure 2: **Total fertility rate by Indian states, 1990–2030.** The median estimates and projections of total fertility rates are shown for the 29 Indian States and Union Territories [9]. The horizontal line is at 2.1, refers to the replacement level of fertility rate.

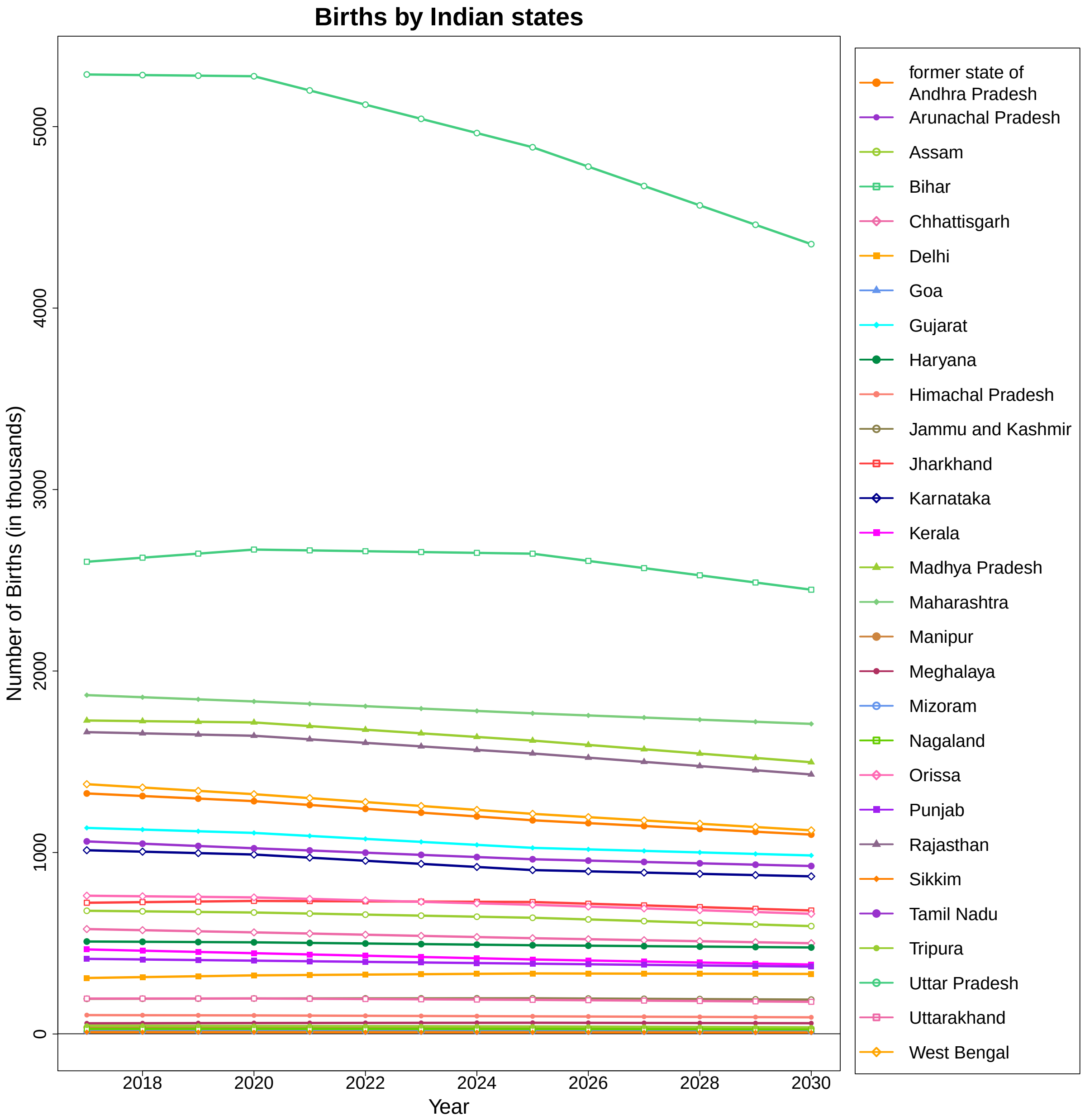

Figure 3: **Number of births by Indian states, 2017–2030.** The median projections of the number of births (in thousands) are shown for the 29 Indian States and Union Territories [9].

Figure 4: **SRB estimates and projections by Indian states, 1990–2030.** The red line and shades are the median and 95% credible intervals of the state-specific SRB. The SRB median estimates before 2017 are from [4]. The green horizontal line refers to the SRB baseline for the whole India at 1.053 [2]. SRB observations used in prior study [4] are displayed with dots and observations are connected with lines when obtained from the same source. Shades/vertical line segments around the data series represent the sampling variability in the series (quantified by two times the stochastic/sampling standard errors). The census data in Jammu and Kashmir is not used to model SRB estimates during 1990–2016 due to its data quality [7]. Blue dashed lines refer to projections from the National Commission on Population, which is based on linear extrapolation [11].

Figure 4 – continued from previous page.

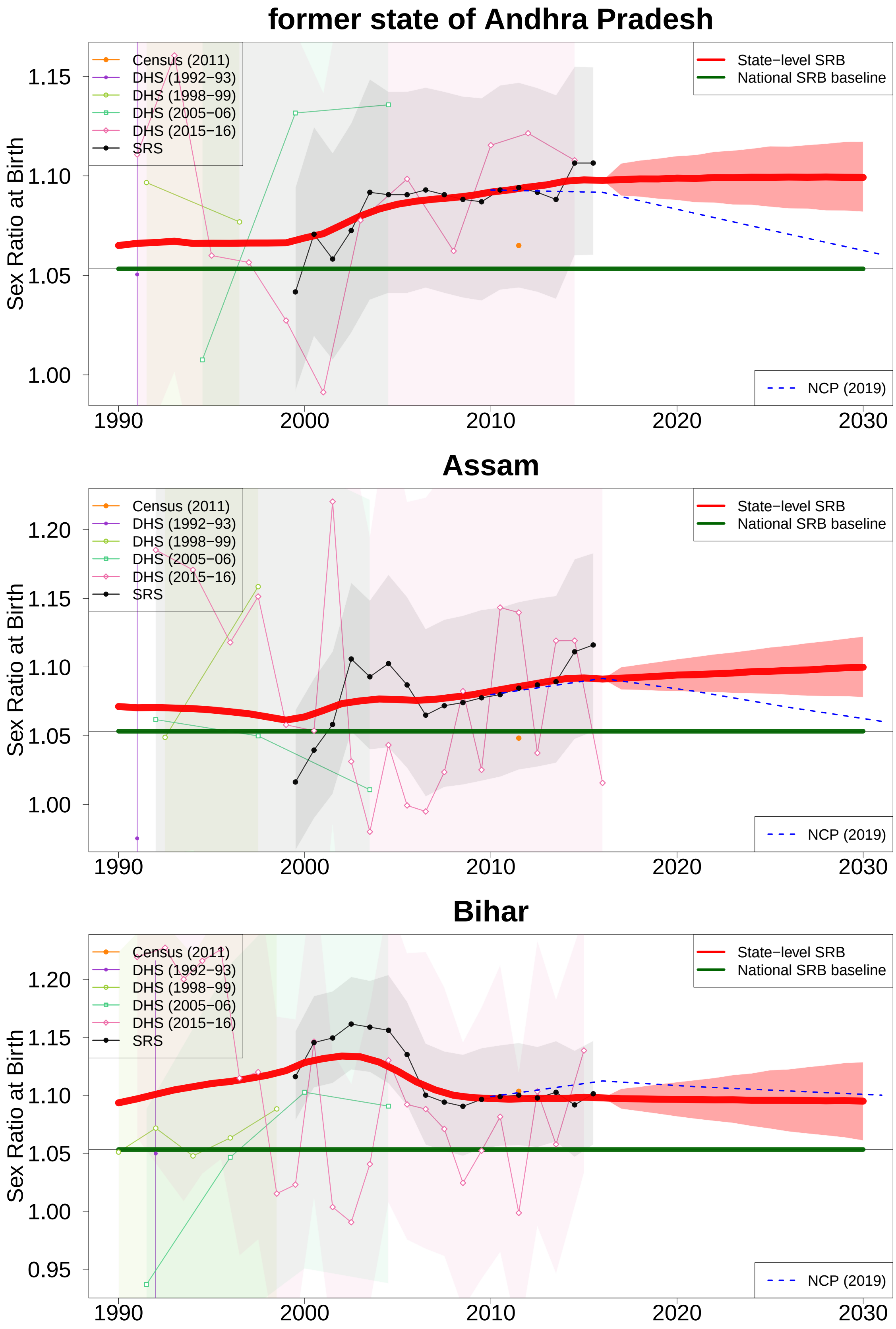

Continued on next page.

Figure 4 – continued from previous page.

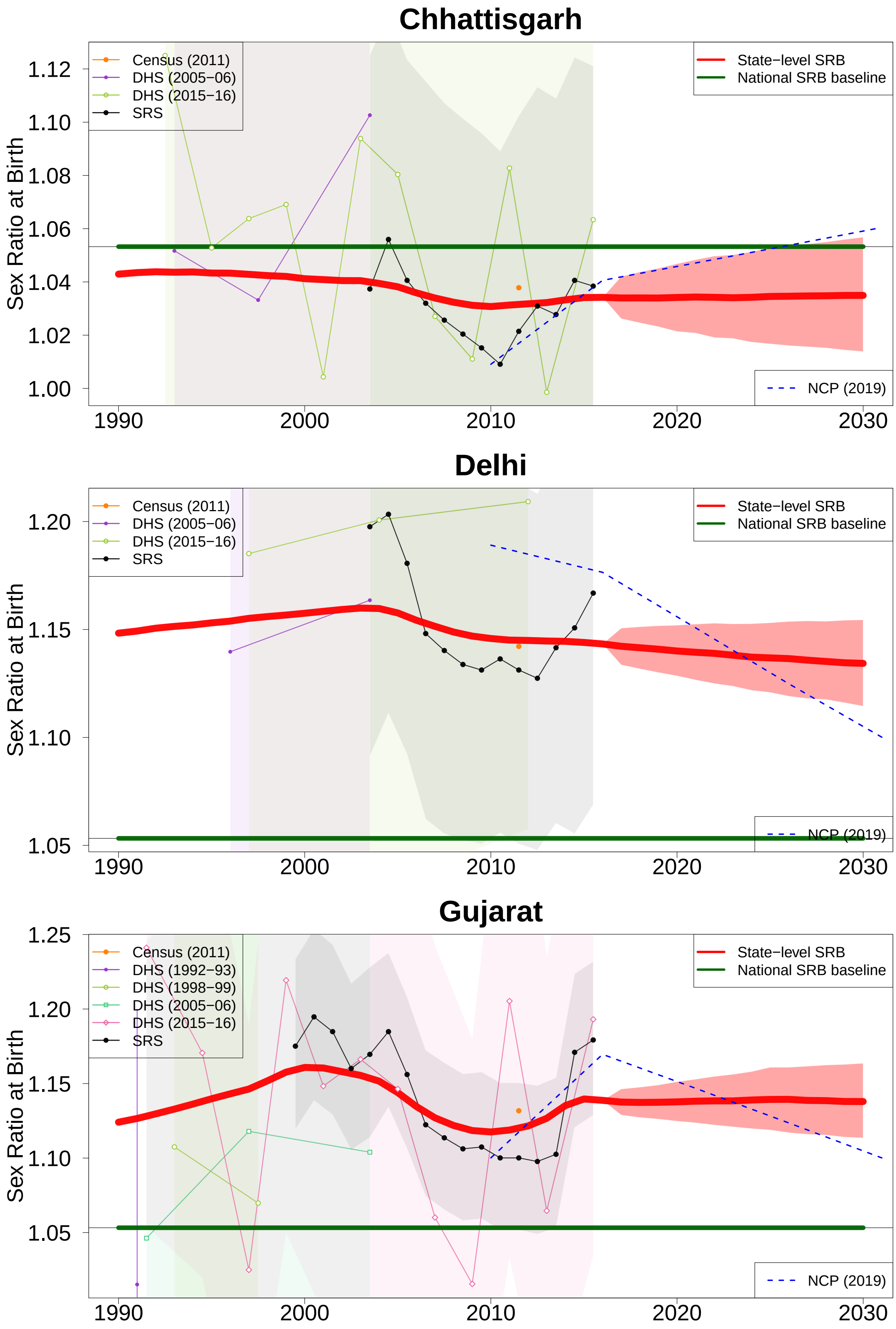

Continued on next page.

Figure 4 – continued from previous page.

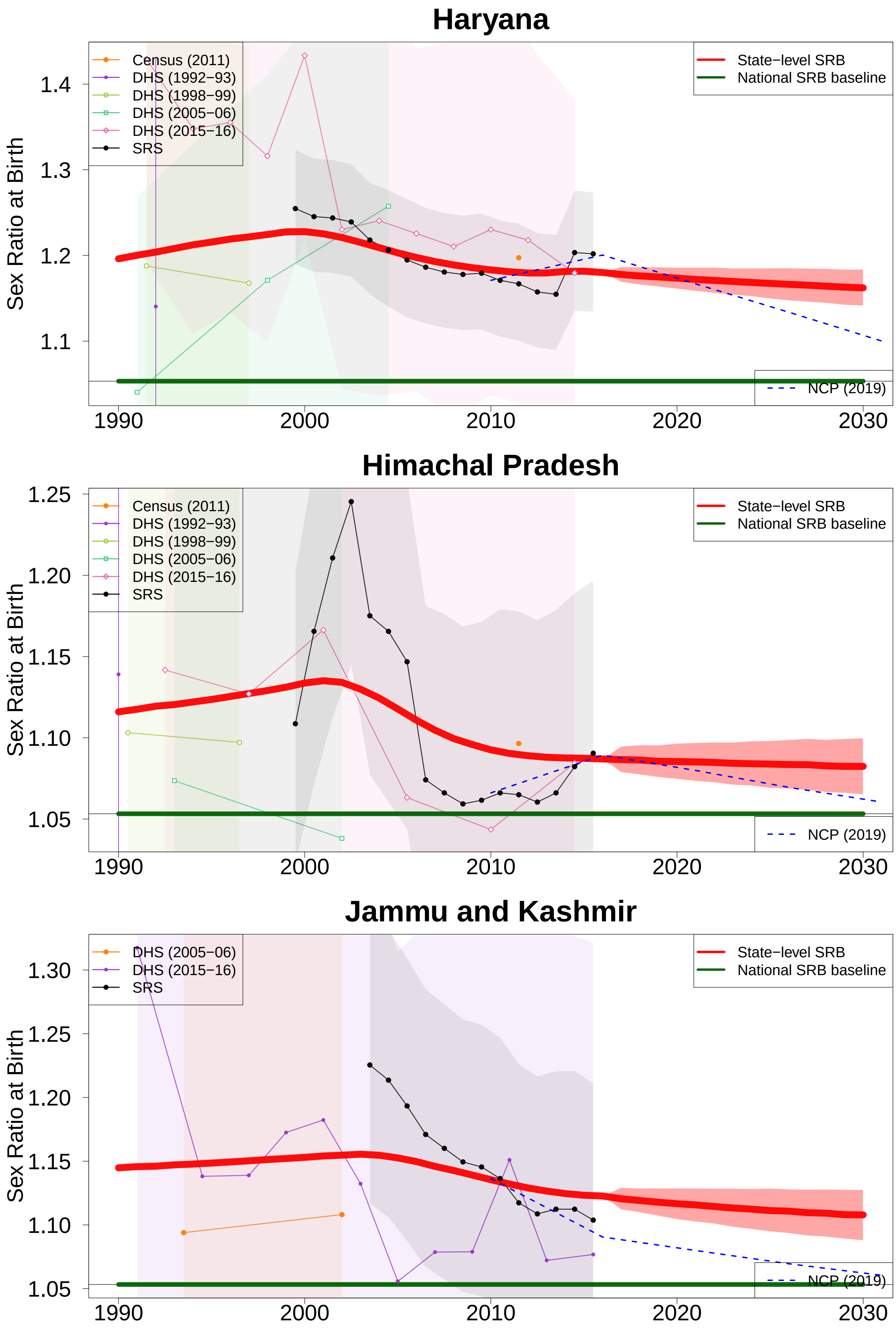

Continued on next page.

Figure 4 – continued from previous page.

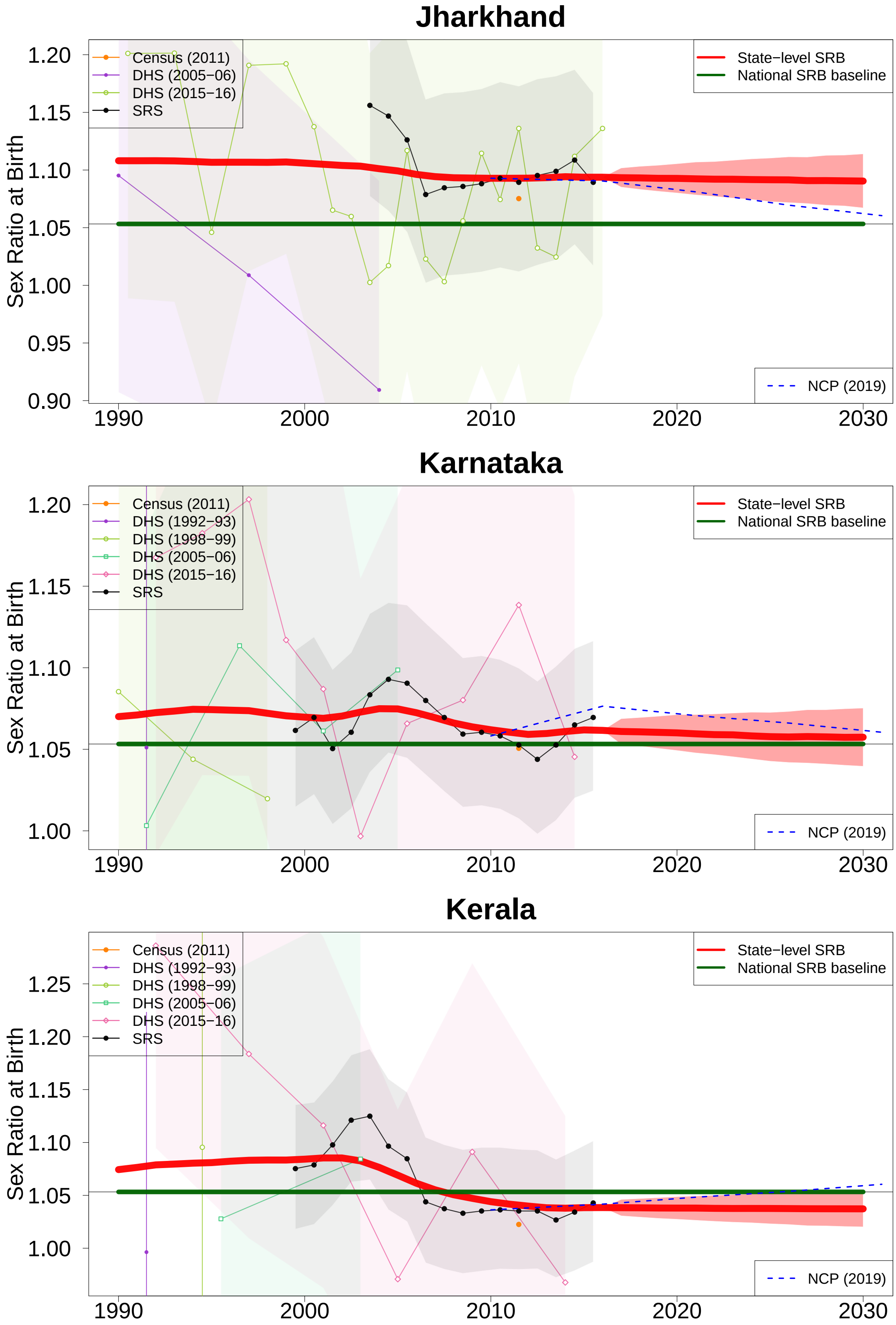

Continued on next page.

Figure 4 – continued from previous page.

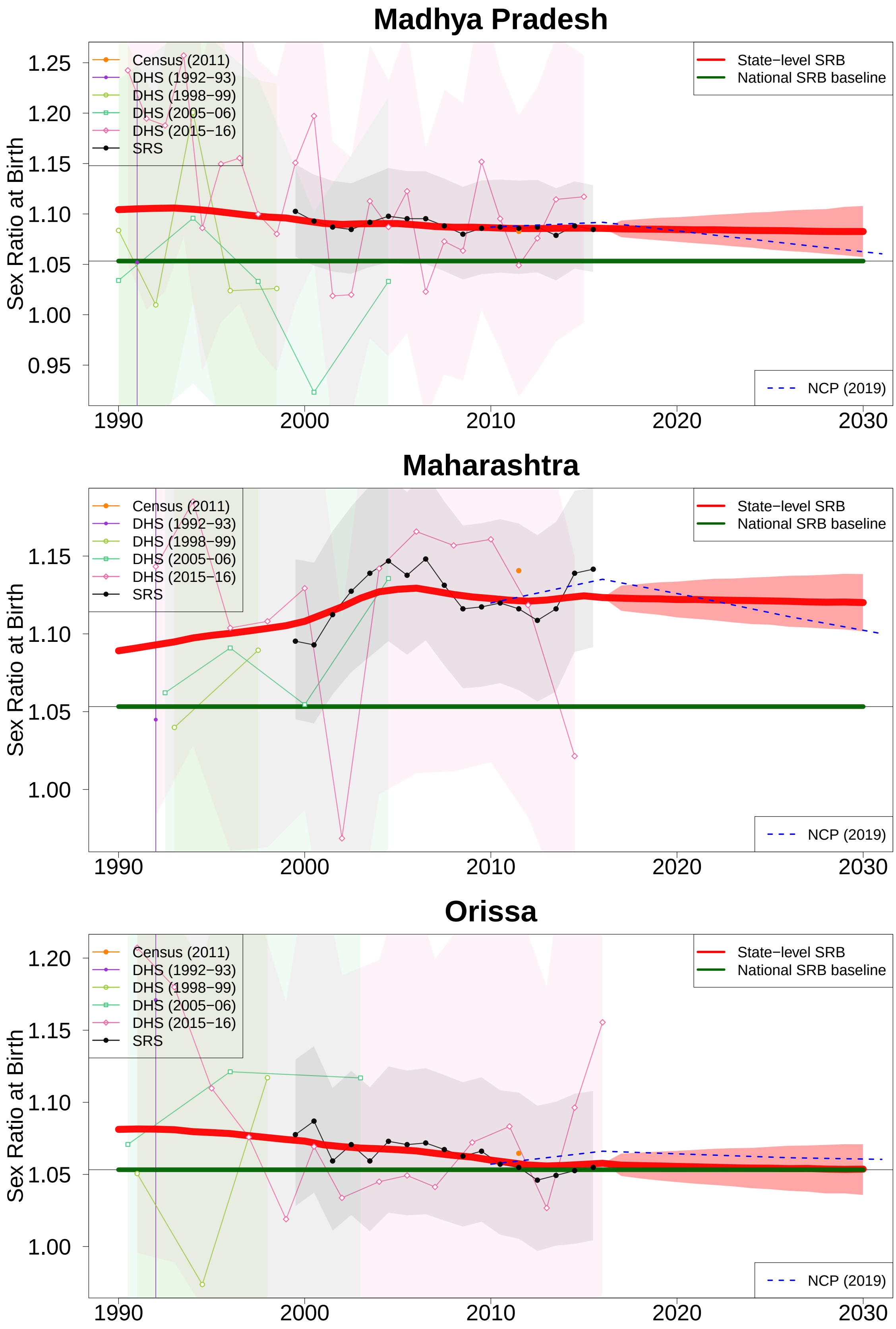

Continued on next page.

Figure 4 – continued from previous page.

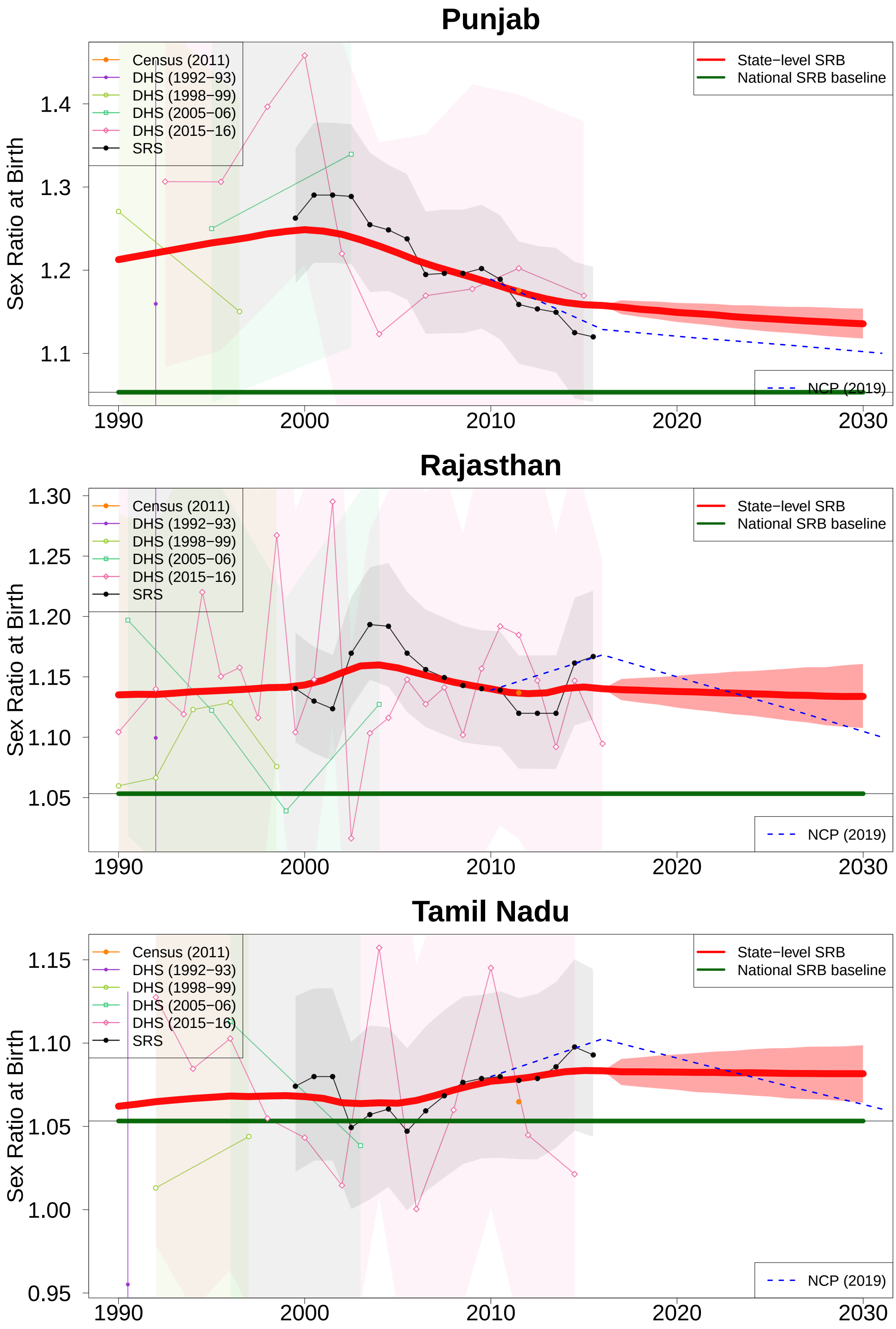

Continued on next page.

Figure 4 – continued from previous page.

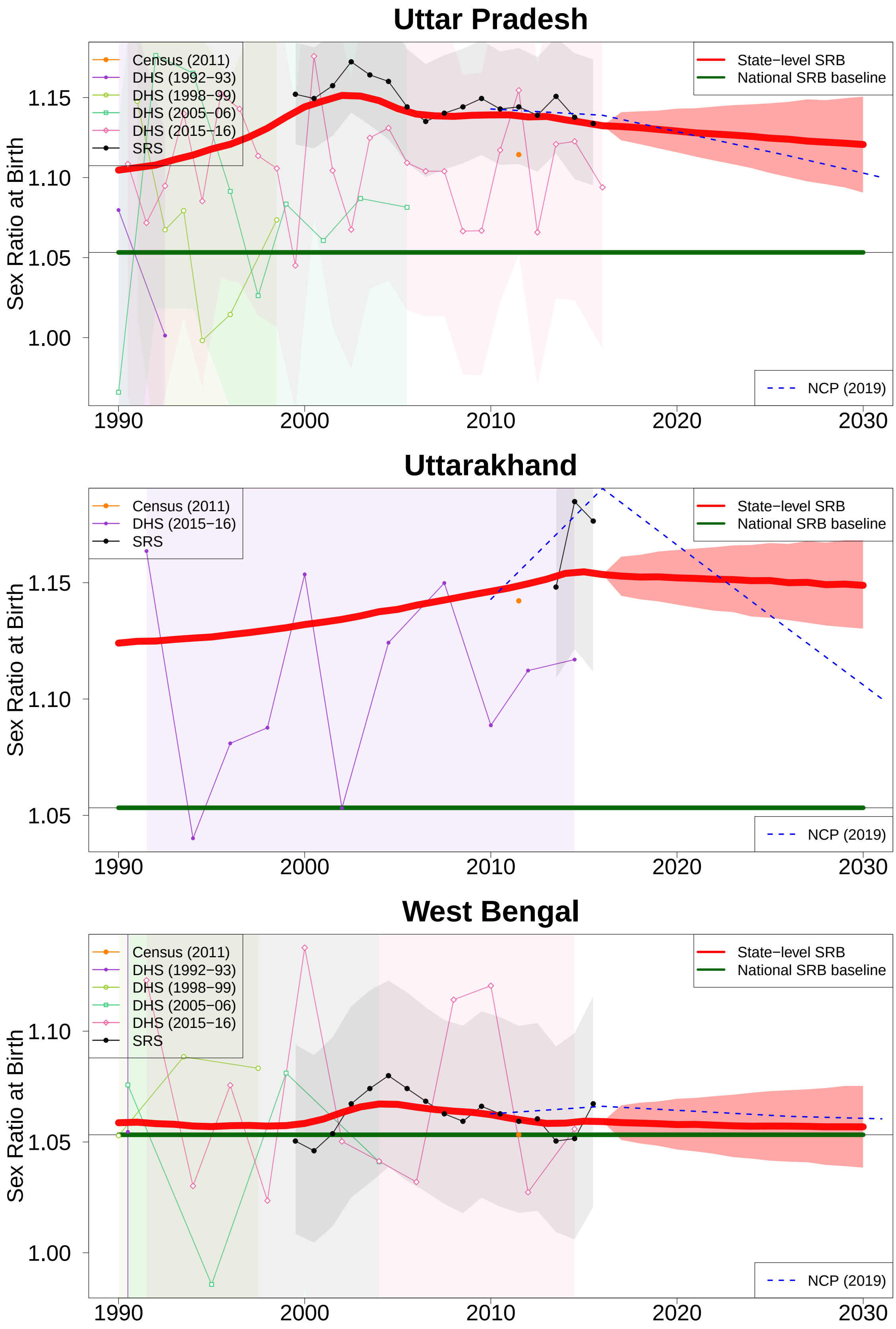

The end of Figure 4.

References

[1] Bongaarts, J. (2013). The implementation of preferences for male offspring. *Population and Development Review*, 39(2):185–208.

[2] Chao, F., Gerland, P., Cook, A. R., and Alkema, L. (2019a). Systematic assessment of the sex ratio at birth for all countries and estimation of national imbalances and regional reference levels. *Proceedings of the National Academy of Sciences*, 116(19):9303–9311.

[3] Chao, F., Gerland, P., Cook, A. R., and Alkema, L. (2019b). Web appendix systematic assessment of the sex ratio at birth for all countries and estimation of national imbalances and regional reference levels. DOI: 10.6084/m9.figshare.12442373. Available at `https://www.pnas.org/content/pnas/suppl/2019/04/10/1812593116.DCSupplemental/pnas.1812593116.sapp.pdf`.

[4] Chao, F. and Yadav, A. K. (2019). Levels and trends in the sex ratio at birth and missing female births for 29 states and union territories in india 1990–2016: A bayesian modeling study. *Foundations of Data Science*, 1(2):177–196.

[5] Gelman, A., Rubin, D. B., et al. (1992). Inference from iterative simulation using multiple sequences. *Statistical science*, 7(4):457–472.

[6] Guilmoto, C. Z., Chao, F., and Kulkarni, P. M. (2020). On the estimation of female births missing due to prenatal sex selection. *Population Studies*, 74(2):283–289.

[7] Guilmoto, C. Z. and Rajan, S. I. (2013). Fertility at the district level in india: Lessons from the 2011 census. *Economic and Political weekly*, 48(23):59–70.

[8] International Institute for Population Sciences (IIPS) and ICF (2017). *National Family Health Survey (NFHS-4), 2015-16: India*. Available at `https://dhsprogram.com/pubs/pdf/FR339/FR339.pdf`. Accessed 17 June 2020.

[9] KC, S., Wurzer, M., Speringer, M., and Lutz, W. (2018). Future population and human capital in heterogeneous india. *Proceedings of the National Academy of Sciences*, 115(33):8328–8333.

[10] Nair, P. S. (2010). Understanding below-replacement fertility in kerala, india. *Journal of health, population, and nutrition*, 28(4):405.

[11] National Commission on Population, Ministry of Health and Family Welfare (2019). *Population Projections for India and States 2011-2036, Report of the Technical Group on Population Projections*. Available at `https://nhm.gov.in/New_Updates_2018/Report_Population_Projection_2019.pdf`. Accessed 17 June 2020.

[12] Plummer, M. (2018). *rjags: Bayesian Graphical Models using MCMC*. R package version 4-8.

[13] Plummer, M., Best, N., Cowles, K., and Vines, K. (2006). Coda: Convergence diagnosis and output analysis for mcmc. *R News*, 6(1):7–11.

[14] Plummer, M. et al. (2003). Jags: A program for analysis of bayesian graphical models using gibbs sampling. In *Proceedings of the 3rd international workshop on distributed statistical computing*, volume 124, page 10. Vienna, Austria.

[15] R Core Team (2019). *R: A Language and Environment for Statistical Computing*. R Foundation for Statistical Computing, Vienna, Austria.

[16] Rue, H., Martino, S., and Chopin, N. (2009). Approximate Bayesian inference for latent Gaussian models using integrated nested Laplace approximations (with discussion). *Journal of the Royal Statistical Society B*, 71:319–392.

[17] Simpson, D., Rue, H., Riebler, A., Martins, T. G., Sørbye, S. H., et al. (2017). Penalising model component complexity: A principled, practical approach to constructing priors. *Statistical Science*, 32(1):1–28.

[18] Sørbye, S. H. and Rue, H. (2014). Scaling intrinsic Gaussian Markov random field priors in spatial modelling. *Spatial Statistics*, 8:39–51.

[19] Su, Y.-S. and Yajima, M. (2015). *R2jags: Using R to Run 'JAGS'*. R package version 0.5-7.
