# Supplementary material for: Probabilistic projection of the sex ratio at birth and missing female births by State and Union Territory in India
Source: PLoS One. 2020 Aug 19;15(8):e0236673. doi: 10.1371/journal.pone.0236673 (PMC7446920; doi:10.1371/journal.pone.0236673)
Supplement: S1 Table — The red numbers at the beginning of each cell refer to the number of States/UTs that fall under each category. (PDF) [file pone.0236673.s002.pdf]

S1 Table. Indian States/UTs classification based on data quality and SRB imbalances. The red numbers at the beginning of each cell refer to the number of States/UTs that fall under each category.

	[21] **With SRS data**	[8] **No SRS data**
[18] **With SRB imbalance**	[16] former state of Andhra Pradesh (including Telangana); Assam; Bihar; Delhi; Gujarat; Haryana; Himachal Pradesh; Jammu and Kashmir; Jharkhand; Madhya Pradesh; Maharashtra; Punjab; Rajasthan; Tamil Nadu; Uttar Pradesh; Uttarakhand	[2] Goa; Manipur
[11] **No SRB imbalance**	[5] Chhattisgarh; Karnataka; Kerala; Orissa; West Bengal	[6] Arunachal Pradesh; Meghalaya; Mizoram; Nagaland; Sikkim; Tripura
